# The potential of rice to offer solutions for malnutrition and chronic diseases

**DOI:** 10.1186/1939-8433-5-16

**Published:** 2012-07-02

**Authors:** Sharifa Sultana Dipti, Christine Bergman, Siti Dewi Indrasari, Theja Herath, Robert Hall, Hueihong Lee, Fatemeh Habibi, Priscila Zaczuk Bassinello, Eduardo Graterol, Julie P Ferraz, Melissa Fitzgerald

**Affiliations:** 1 Grain Quality and Nutrition Centre, International Rice Research Institute (IRRI), DAPO, 7777 Metro Manila, Philippines; 2 Department of Food and Beverage, University of Nevada-Las Vegas, Las Vegas, NV 89154 USA; 3 Indonesian Center for Rice Research (ICRR), BB Padi, Jl. Raya 9, Sukamandi, Subang, 41256 Jawa Barat Indonesia; 4 Industrial Technology Institute, Colombo 7, Bauddhaloka Mawatha, 363 Sri Lanka; 5 Plant Research International, PO Box 98, 6700AB Wageningen, The Netherlands; 6 Centre for BioSystems Genomics, P.O. Box 98, 6700AB Wageningen, The Netherlands; 7 Faculty of Agriculture and Food Sciences, Universiti Putra Malaysia, Nyabau Road, 97000 Bintulu Sarawak, Malaysia; 8 Rice Research Institute of Iran (RRII), Km5 Tehran Rd, 41996-13475 Rasht, I.R Iran; 9 Embrapa Arroz e Feijão, Rodovia GO-462, Km 12, Zona Rural C.P. 179, Santo Antônio de Goiás, GO 75375-000 Brazil; 10 Fundación para la Investigación Agrícola DANAC, Apartado Postal 182, San Felipe, Estado Yaracuy Venezuela; 11 Institute of Science, Diabetes Foundation Marikina, Philippines, Healthserve Hospital, and Calamba Doctors Hospital, Laguna, Philippines; 12 International Network for Quality Rice, Metro Manila, Philippines; 13 Grain Quality and Nutrition Centre, International Rice Research Institute (IRRI), DAPO 7777 Metro Manila, Philippines; 14 School of Agriculture and Food Science, University of Queensland, St Lucia, 4072 Australia

## Abstract

It is internationally accepted that malnutrition and chronic diseases in developing countries are key limitations to achieving the Millennium Development Goals. In many developing countries, rice is the primary source of nutrition. In those countries, the major forms of malnutrition are Fe-induced anaemia, Zn deficiency and Vitamin A deficiency, whereas the major chronic disease challenges are Type II diabetes, cardiovascular disease and some cancers. There is a growing corpus of evidence regarding both limitations and opportunities as to how rice could be an effective vehicle by which to tackle key nutrition and health related problems in countries with limited resources. Rice breeding programs are able to focus on developing new varieties carrying enhanced amounts of either Fe, Zn or beta-carotene because of large public investment, and the intuitive link between providing a mineral/vitamin to cure a deficiency in that mineral/vitamin. By contrast, there has been little investment in progressing the development of particular varieties for potential impact on chronic diseases. In this review article we focus on the broad battery of evidence linking rice-related nutritional limitations to their impact on a variety of human health issues. We discuss how rice might offer sometimes even simple solutions to rectifying key problems through targeted biofortification strategies and finally, we draw attention to how recent technological (−omics) developments may facilitate untold new opportunities for more rapidly generating improved rice varieties specifically designed to meet the current and future nutritional needs of a rapidly expanding global population.

**Electronic supplementary material:**

The online version of this article (doi:10.1186/1939-8433-5-16) contains supplementary material, which is available to authorized users.

## Review

### Background

As we enter the second decade of the 21^st^ century, experts agree that the world faces three major global health challenges. The first is completing the work to meet the millennium development targets of decreased malnutrition and infectious disease. The second is the alarming increase in the incidence of chronic diseases like heart disease, Type II diabetes, obesity, and cancers in developing nations. The third is a consequence of globalisation whereby traditional diets are being replaced or supplemented with nutritionally compromised fast-foods. Nutrition is a feature of each of these challenges, and in a sad twist of irony, developing countries, where rice is the staple, are the hardest hit by all three of these global challenges.

Over the past decade, rice improvement programs have included key nutritional targets in their breeding programs, attempting to meet specific targets for Fe, Zn and pro-vitamin A content. During this time, significant progress has been made in understanding both ways to increase the micronutrient content of the polished and unpolished rice, and the limitations to achieving those targets in conventional breeding programs. However, the potential of rice to contribute to the prevention or management of chronic diseases is not so widely recognised, and research aimed to quantify that potential receives a fraction of the public funding of rice and malnutrition, despite the massive and growing problem of chronic disease that prevails in all rice-consuming countries ([Nugent 2008]). Perhaps this is because the association between compounds in rice grains and chronic diseases is more complex and less intuitive than, for example, increasing Zn content of grains to address Zn deficiency. A second reason could be limitations in the detection and identification of relevant grain constituents, meaning that phenotyping tools are not available for breeding objectives. The previous decade has borne witness to breath-taking technological advances, which should enable research to progress more rapidly both in identifying grain constituents, and determining the impact of these in the prevention or management of chronic diseases.

This review will focus on the nutritional potential of grains of both polished and unpolished rice, for the most pressing issues of malnutrition and chronic diseases in rice-consuming countries. We discuss opportunities and obstacles, and identify roles that rice might play in health and nutritional impact, and patterns of rice consumption that could contribute to solutions for the grand challenges to global health.

## Malnutrition

### Malnutrition in rice-consuming populations

Iron deficiency anaemia is a worldwide public health problem, with global prevalence estimated at 24.8% (95% CI: 22.9–26.7) ([Shaw and Friedman 2011]). It occurs when the concentration of haemoglobin (Hb) falls below 11 g/dl in pregnant women, 12 g/dl in non-pregnant women aged 15–49, and 11 g/dl in children under five. Anaemia can cause maternal mortality associated with childbirth. In adults it lowers work performance, and it has been linked with reduced immune competence ([Shaw and Friedman 2011]). The majority of the disease burden is shouldered by developing countries with high levels of rice consumption. The highest prevalence is found in Africa, the Middle East, Central, South and South-East Asia, and areas of Latin America, where two thirds of children under five, and almost 50% of women are anaemic ([WHO 2008]). One reason that Fe-deficient anaemia is widespread amongst rice-consuming countries is because of the low concentration of Fe in polished rice, like other starchy staples, combined with the inability of poor people to supplement the staple with other foods rich in micronutrients. A survey of 56 varieties showed that the average Fe content of the polished grains was 4.3 ppm ([Bounphanousay 2007]); however, the biological availability of Fe from polished rice is low ([Shaw and Friedman 2011]).

The first cases of zinc deficiency were described in the Middle East the 1960s, and attributed to the consumption of diets high in anti-nutritional factors ([Prasad et al. 1963]). Zn deficiency is now recognised as one of the five major factors contributing to disease burden in developing countries ([WHO 2002]). Zn deficiency leads to decreased neuropsychological function, it contributes to childhood mortality by increasing the incidence and severity of acute and chronic diarrhoea, and in pregnant women it leads to difficulties in childbirth, retarded foetal growth, and foetal abnormalities ([Prasad 2003];[Tamura and Goldenberg 1996]). The prevalence of Zn deficiency in developing countries is similar to that of Fe deficiency, since the same dietary pattern, a reliance on polished rice with minimal dietary diversity, contributes to both ([Black et al. 2008]). In Central, South and South-East Asia and sub-Saharan Africa, stunting due to Zn deficiency affects 40% of preschool children ([Hotz and Brown 2004]), and 82% of pregnant women ([Bhutta and Haider 2009]). Most Latin Americans living in poverty consume a diet rich in cereals and beans, and low in animal products ([Berne Pena et al. 2008]), and therefore record high levels of Zn deficiency ([Amaya et al. 2002];[WHO 2008]).

Vitamin A deficiency (VAD) is as a public health problem among preschool-aged children in 118 developing countries around the globe ([West 2002]). Vitamin A deficiency occurs when serum retinol is less than 0.7 μmol/l, or less than 20 μg/dl in children below 6 years. The prevalence of VAD among school-aged children (5-15y) in Latin American, South and Southeast Asian countries varies from 6% in Sri Lanka to 36% in El Salvador ([Amaya et al. 2002];[West 2002]).

Vitamin A plays a major role in phototransduction, and deficiency leads to xerophthalmia followed by complete blindness ([Mason et al. 2005]). Beta-carotene is the precursor of Vitamin A. In a survey of 3000 varieties of rice, only 20 varieties were found to contain β-carotene ([Fitzgerald 2007]). The amount of β-carotene detected in those 20 was less than 0.2 ppm, and it was found only in the bran layer; no β-carotene was detected in the polished grains of any of the varieties ([Fitzgerald 2007]). Therefore people deriving most of their calories from polished rice are at the highest risk of VAD, since other staples, such as certain varieties of wheat, maize and orange-fleshed sweet potatoes contain β-carotene.

### Solutions to malnutrition through rice

In the context of such widespread malnutrition in the world’s major rice consuming countries, agricultural strategists recognised a potential role for rice, if breeding programs could elevate the micronutrient levels in rice ([Bouis and Hunt 1999]). This led to a paradigm shift in breeding programs, as selection for nutritional traits commenced, with the objectives of elevating Fe and Zn, and incorporating β-carotene into polished grains. This program was first launched under the umbrella of the Consultative Group for International Agricultural Research (CGIAR) Micronutrient Program, which then underwent reform in 2004 to become Harvestplus, an autonomous organisation that sets the targets and coordinates the multilateral efforts to increase Fe, Zn and β-carotene in staple crops, including rice (http://www.harvestplus.org).

**Figure 1:**
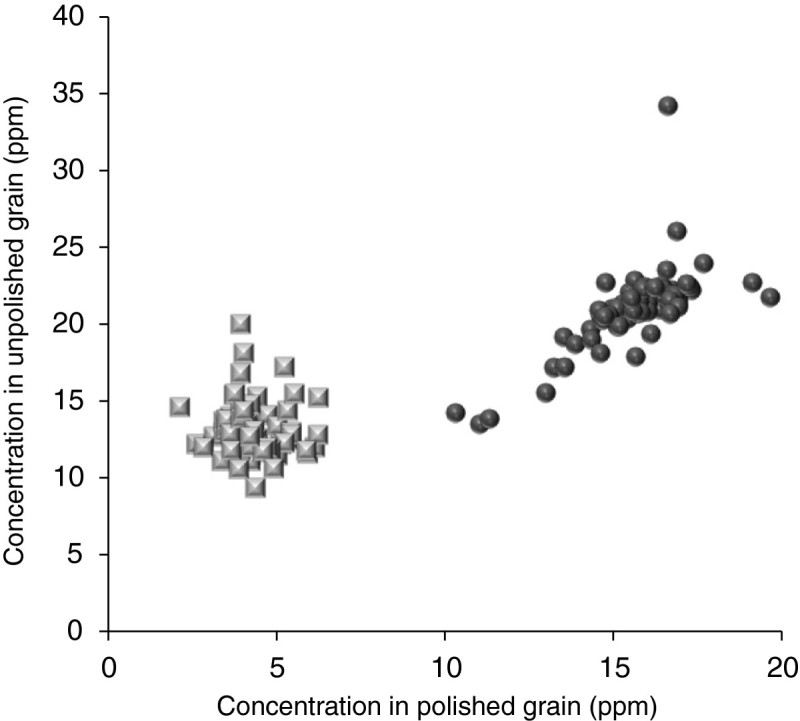
**Fe (squares) and Zn (circles) content of unpolished and polished rice of seventy varieties of rice.**

The minerals in unpolished rice of 60 popular varieties (Table1) show that there is less than 10% variability for all minerals except Zn, Mn, and Cu. By exploring wider sources of diversity within the species, significantly more variability was found for the amount of Fe and Zn in unpolished rice ([Gregorio 2002]). However it is not always possible to extrapolate data from unpolished rice to white, polished rice. By replotting data of 70 varieties of unpolished rice and the same varieties polished to a degree of milling of 10% ([Bounphanousay 2007]), it can be seen that the Fe content of all the polished rices clusters around 4 – 5 ppm, despite variation in Fe before removal of the bran layer (Figure1). By contrast, the Zn content of the unpolished grains shows an association with the Zn content when the grains are polished, and also shows a range in Zn contents of polished rice from 10 – 20 ppm (Figure1).

**Table 1:** **Amount of selected micro and macro minerals in unpolished grains of 60 popular rices from South-East Asia (Data from Indrasari 2002)**

	Ca	P	Mg	K	S	Fe	Zn	Mn	Cu
**Amount (ppm)**	92.44	3720.59	1488.82	2823.53	1309.12	11.69	23.89	32.51	3.07
**Standard deviation**	14.13	243.43	103.59	264.07	111.37	1.71	3.96	7.25	1.04

In the past ten years, significant international effort has been expended to search the diversity of the species for elevated Fe and Zn in the polished grain ([Hunt et al. 2002];[Jiang et al. 2007];[Liang et al. 2007];[Liang et al. 2008];[Liu et al. 2004];[Ma et al. 2005];[Tucker 2003];[Vasconcelos et al. 2003];[Wang et al. 2004];[Welch and Graham 2004]). As suggested by Figure1, variation for Zn content was discovered, and focussed selection and careful phenotyping has enabled rice improvement programs to release varieties with elevated Zn ([Virk and Barry 2007]). Models using the levels of Zn that can now be achieved in polished rice suggest that the increase in Zn will lead to a significant decrease in the prevalence of Zn deficiency in both adults and children in rural Bangladesh ([Arsenault et al. 2010]).

After searching widely through the diversity of the species, and its wild relatives, rice improvement programs have not found variation in the endosperm for Fe. This suggests that there is either physiological regulation of the Fe that exchanges from the maternal to the filial tissues; or no biological reason, therefore mechanism, for Fe to accumulate in the endosperm. Rice improvement programs have therefore concluded that elevating Fe in the grain can only be achieved by the use of transgenic techniques ([Johnson et al. 2011]).

Transgenic technology successfully elevates Fe in the grain, suggesting no physiological barrier regulating it. In one study, a ferritin gene from soybean was expressed in the endosperm, leading to the accumulation of Fe ([Qu et al. 2005];[Vasconcelos et al. 2003]). Another study pyramided a ferritin gene from common bean into the grain to increase Fe, with a phytase gene from *Aspergillus* to increase bioavailability of the Fe ([Lucca et al. 2002]). More recently, one study showed a significant increase in Fe content of polished grains by expressing a nicotianamine synthase (*NAS*) gene from barley in the rice endosperm ([Lee et al. 2009]). In another study, the *NAS* genes from rice, *OsNAS1*, *OsNAS2* and *OsNAS3*, that usually express in roots and shoots, were expressed in the endosperm leading to levels of Fe in polished grains that meet and exceed the Harvestplus targets ([Johnson et al. 2011]). Taken together, these studies show that it is possible for iron to enter the endosperm in different forms, and the studies all indicate that a mechanism to import Fe into the endosperm has simply not previously evolved. The ability for different forms of Fe to accumulate provides options to maximise its bioavailability.

The small amount of iron that occurs naturally in the grain and the aleurone layers of the endosperm was unable to reverse Fe-induced anaemia in women ([Haas et al. 2005]). Transgenic rice with the ferritin gene from soybean was able to reverse anaemia in rats with the same efficiency as FeSO_4_ ([Murray-Kolb et al. 2002]), but the same rice had no effect on iron status of piglets ([Schaffer et al. 2004]). These contradictory findings suggest that bioavailability of ferritin is complex. By contrast, the Fe chelated in nicotianamine was found to be bioavailable to humans ([Zheng et al. 2010]). This suggests an argument for testing the most powerful gene for elevating endosperm Fe, *OsNAS2* ([Johnson et al. 2011]), in other genetic backgrounds, to test (i) if the phenotype is the same in different genetic backgrounds, (ii) for any negative effect on grain yield and grain quality, and (iii) whether the next generation of seedlings, nourished by the endosperm, suffers any effect due to the presence of the iron in the endosperm. Varieties expressing *OsNAS2* in the endosperm possibly offer a solution, other than by reason of bioavailability, to reversing Fe-anaemia. The other transgenic examples use genes from other species to accumulate Fe, whereas those with *OsNAS2* in the endosperm accumulate Fe using a gene that naturally occurs in rice. Transgenic technology was used only to change the expression pattern of that gene. This difference in the degree of transgenic technology, where transgenic techniques were only used to change where the gene expresses in the plant, could ease the road to deregulation and the grains might be acceptable to a wider group of consumers.

A solution to VAD is being tested using transgenic technology to insert the genes necessary to enable the rice grain to accumulate β-carotene, creating Golden Rice ([Beyer et al. 2002];[Grusak 2005]). The transgenes have recently been crossed into popular and high-yielding varieties using conventional breeding techniques (http://irri.org/news-events/hot-topics/golden-rice). Recently, a preliminary feeding trial in the US showed that the β-carotene from Golden Rice is efficiently converted to retinol ([Tang et al. 2009]). However, there is very little other information in the public arena about the effect of the transgenes on nutritional, sensory and postharvest quality of the grains of Golden Rice. The high bioavailability, relative to some other sources, was ascribed to the simple starch matrix of the rice ([Tang et al. 2009]). In carrots, β-carotene is located in crystalline chromoplasts where it is less bioavailable than the β-carotene from mangoes, which is held in lipid droplets ([Brackmann et al. 2011]). The location of β-carotene in rice grains is unknown, but its high bioavailability suggests that it is not in chromoplasts. This could also mean that the β-carotene is susceptible to auto-oxidation ([Ramakrishnan and Francis 1979]). After harvest, rice is usually dried to about 12% moisture for storage. In a starch system containing crystalline β-carotene, and stored at room temperature for four weeks at 11% moisture, a 30% loss in β-carotene, due to auto-oxidation was found ([Ramakrishnan and Francis 1979]). Presumably the creators of Golden Rice have achieved a balance between bioavailability and loss due to auto-oxidation, in order to maximize the potential impact on the consequences of VAD, and have fully characterized any oxidation products that could accumulate in grains. Wider performance testing, including yield trials as well as clinical trials with VAD-deficient target populations in developing countries will be conducted in the near future to verify both bioavailability and potential health benefits of Golden Rice (http://irri.org/news-events/hot-topics/golden-rice). Assuming that all regulatory requirements will be met, IRRI projects that Golden Rice may be ready for release in 2013 (http://www.irri.org).

## Chronic diseases

### Chronic diseases in rice consuming countries

**Figure 2:**
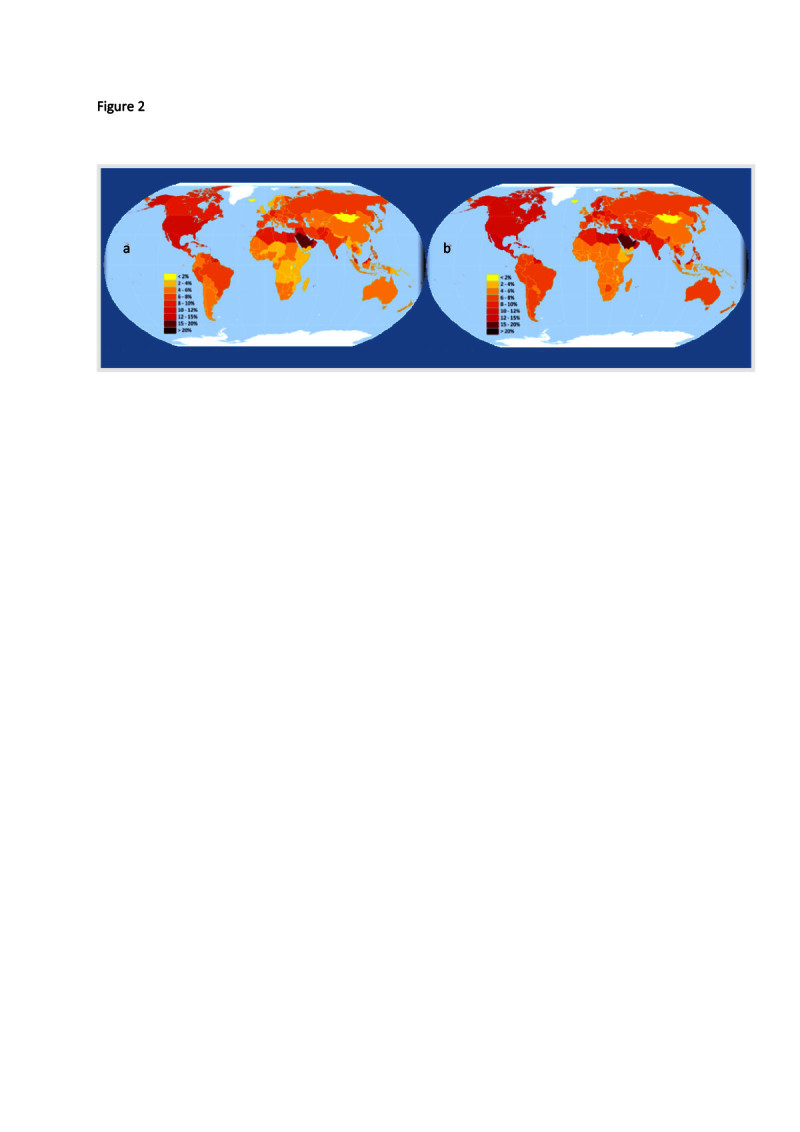
**Global distribution of the prevalence of type II diabetes in 2010 (a) and project in 2030 (b).** Replotted from the Diabetes Atlas data (www.idf.org/diabelesatlas).

The total number of people with Type II diabetes mellitus (DM) is projected to rise from 171 million in 2000 to 366 million in 2030 ([Wild et al. 2004]). Figure2 shows the prevalence of Type II DM in each country in 2010, and the projected distribution by 2030 ([Shaw et al. 2010]). Saudi Arabia and North America have the highest prevalence (Figure2). By 2030, a significant increase in prevalence is seen for Latin America, Africa, and South and Southeast Asia ([Shaw et al. 2010]). One of the major risk factors is that developing countries are changing their eating habits ([Misra et al. 2010]), leading to an obesity epidemic, termed the nutrition transition ([Astrup et al. 2008];[Hossain et al. 2007];[Yoon et al. 2006]). In many developing countries, low fat diets are being replaced by fast foods that are high in fat, and this is leading to significant increases in obesity ([James 2008]). The increase in the proportion of the population with obesity is one of the greatest risk factors for diabetes, coronary disease and some cancers ([Kopelman 2000]). Coupled with population growth, rice consuming countries are heading towards a major public health crisis, with significant financial risk at both the household and national levels.

Each year, an estimated 2.5 billion cases of diarrhoea occur among children under five years of age ([Zaidi et al. 2004]). Significantly, the median incidence of diarrhoeal diseases in children under five in developing countries has changed little since the early 1990s ([Jamison et al. 1993];[Parashar et al. 2003]). More than half of these cases are in Africa and South-East Asia, where bouts of diarrhoea are more likely to result in death or other severe outcomes, such as significant loss of vitamins and minerals ([Udomkesmalee et al. 1990]). By placing these figures and trends in the context of the United Nations Millennium Development Goal 4, which aims to reduce childhood mortality by two-thirds between 1990 and 2015, it becomes clear that many countries in South-East Asia might not meet this target ([You et al. 2010]). In addition, these data provide an illustration of the vicious cycle of malnutrition, where chronic diarrhoea could prevent children from reaping nutritional benefits of rice varieties, developed especially to address malnutrition, with elevated concentrations of vitamins and minerals.

In 2000, more than half the 16.7 million world deaths from cardiovascular diseases were in developing countries, ([WHO 2001]) many of these in South and South-East Asia. Coronary deaths in India are expected to double over the next 20 years ([Ghaffar et al. 2004]), and they reached 2 million in 2010. Cardiovascular diseases were the leading cause of death Malaysia ([Statistics 2010]), which is mainly due to hypercholesterolaemia and hypertension ([Statistics 2006];[Yunus et al. 2004]). In 1998, the cause of death from cardiovascular disease in Bangladesh was 12.5%, Bhutan 14.8%, India 13.0%, Indonesia 23.2%, Maldives 24.8%, Myanmar 12.7%, Nepal 1.9%, Sri Lanka 20.1%, and Thailand 17.1% ([WHO 2002]).

Cardiovascular disease is a far greater public health problem in developing countries than previously realized, particularly in younger people, according to a report that combined population estimates for five lower to middle income countries with current death rates and workforce data to calculate the effects of cardiovascular disease on society and on the workforce. A conservative estimate showed that at least 21 million years of future productive life were lost each year in the five countries because of cardiovascular disease. Future predictions were even more disturbing, with the number set to rise to 34 million years of life lost by 2020 ([London 2004]).

The cost of managing cardiovascular diseases has been increasing in several countries and regions of the world ([Leal et al. 2006]). One study estimated the burden of cardiovascular diseases in 24 countries of the European Union, and reported the financial burden of cardiovascular diseases was €169 billion per year, with direct healthcare costs accounting for 62% of the cost, followed by costs of informal care, and indirect costs associated with loss of production due to early death, and the loss of productivity due to morbidities ([Leal et al. 2006]).

### Potential solutions to chronic diseases from rice

Whole-grains are the unpolished version of cereal grains, consisting of the germ, bran, and endosperm; while polished grains lack both the germ and bran. The scientific community generally considers whole-grain foods to include those that contain the same amount of germ and bran that would typically be found in the unprocessed grain ([Seal et al. 2006]). By this definition, whole-grain rice, whether consumed intact or pulverised into flour, is a whole-grain food. Consequently, epidemiological studies designed to identify dietary patterns associated with reduced incidence of chronic disease typically classify unpolished, or brown, rice as a whole-grain ([Jacobs et al. 2007];[Wang et al. 2007]). There is a significant corpus of research describing components in whole-grain rice which have potential for nutritional impact ([Amissah et al. 2003];[Eggum 1979];[Goffman et al. 2003];[Rukmini and Raghuram 1991];[Shen et al. 2009];[Storck et al. 2005]).

Whole-grains are hypothesised to contribute positively to human health due to their fibre, minerals (Table1), vitamins (e.g., vitamins B, D and E), phenolic compounds, phytoestrogens (lignans), and other phytochemicals ([Slavin et al. 1999]). These compounds may influence biological functions individually or synergistically. Whole grain rice contains similar types of compounds to other cereal grains albeit with a few unique types and in unique percentages.

Epidemiological studies suggest consuming whole-grains provides a protective effect against several chronic diseases. Whole-grain rice contains unique types and amounts of some phytochemicals such as the gamma-oryzanol and tocotrienol fractions. Numerous cell culture, animal, and human-based studies have demonstrated the potential health benefits of consuming whole-grain rice and some of its phytochemical fractions. The majority of these studies have focused on the reduction of risk factors for cardiovascular disease ([Cicero and Derosa 2005];[Hallfrisch et al. 2003]), Type II diabetes ([King et al. 2008];[Topping 2007];[Zhang et al. 2010]), and several cancers ([Williams and Hord 2005]). By far, the strength of the evidence lies in there being a positive relationship between whole grain rice consumption and reduced risk of disease. Some of the studies supporting these associations, however, have dealt with specific constituents in rice bran and endosperm, and have involved administration of fractions at higher intakes than would be practical from consuming whole grain rice. However, it is possible that these fractions may impact biological targets synergistically and thus exert much higher chemo-preventive efficacy than that found for individual compounds. This possibility may be even more likely when whole grain rice is consumed as part of the daily diet over a long period of time.

The bran components with potential nutritional value include the vitamin E complex of unpolished rice, which is unusually high, ranging from 179–389 mg/kg bran, with an average of 72.5% of the isomers being tocotrienols ([Bergman and Xu 2003]). Other cereals contain much lower average amounts of Vitamin E compared with rice: wheat 23 mg/kg, barley 8 mg/kg, spelt 18.1 mg/kg, and rye 11.9 mg/kg ([Nielsen and Hansen 2008]). The lipid content of rice bran is also high in comparison to other grains. Its primary unsaturated fatty acids are oleic, linoleic and alpha-linoleic, while its primary saturated fatty acids include palmitic and stearic acids. The non-saponifiable fraction of rice bran oil contains tocotrienols, tocopherols, phytosterols, gamma-oryzanol compounds, policosanols, and saponines. Each of these phytochemical fractions consists of several compounds. For example, the gamma-oryzanol fraction is composed of ferulic acid esters of triterpene alcohols. The three primary compounds are cycloartenyl ferulate, 24-methylenecycloartanyl ferulate and campesteryl ferulate ([Xu and Godber 1999]), and there are at least seven more compounds in that fraction ([Akihisa et al. 2000];[Xu and Godber 1999]). Rice bran also contains phenolic compounds which reportedly vary a great deal in quantity and type across different cultivars ([Goffman and Bergman 2002];[Goffman and Bergman 2004]).

#### Cardiovascular disease and whole-grain rice

Studies on the potential health promoting properties of rice on cardiovascular diseases began more than four decades ago ([Nakamura 1966]). These studies report positive effects of whole-grain rice (and several of its fractions) consumption on cardiovascular disease risk factors, such as hypertension and cholesterol, using rodents, rabbits, non-human primates, and humans ([Cicero and Derosa 2005]). The association between whole-grain consumption and protection against heart disease and stroke is considered unequivocal by many, but the exact mechanism is not clear ([Flight and Clifton 2006]).

#### Hypertension

Hypertension is a significant risk factor for coronary disease. The Dietary Approaches to Stop Hypertension (DASH) diet recommends those with hypertension to increase consumption of whole-grains ([Lochner et al. 2006]). These recommendations are based on the findings of cross-sectional studies examining the correlations between lifestyle and the development of cardiovascular disease. Conclusions from studies examining the specific effect of whole grain consumption on blood pressure, however, have been inconsistent ([Davy et al. 2002];[Pins et al. 2002]). Many of these studies did not control dietary composition except for whole-grain content and some used whole-grain fractions as opposed to whole-grain foods. By including whole-grain rice as a focus, the design of many of the previous studies evaluating whole-grain consumption and hypertension improved ([Hallfrisch et al. 2003]). Non-hypertensive men with elevated plasma cholesterol levels were fed an American Heart Association Step 1 diet with or without inclusion of unpolished rice/whole wheat, barley, or a combination in a Latin square design. Also controlled were levels of protein, calcium, magnesium, sodium, and potassium in the diets. Systolic, diastolic, and mean arterial blood pressures were reduced in those who consumed soluble fibre from barley or insoluble fibre from unpolished rice and whole wheat, and consumption of the Step 1 diet without the whole-grain component did not have any effect on blood pressure ([Hallfrisch et al. 2003]).

#### Serum lipid levels

A large number of animal, nonhuman primate, and human-based studies provide strong evidence that rice bran and its fractions lower serum cholesterol and triglyceride levels ([Cicero and Derosa 2005]). One study, using 18 humans with moderately-high blood cholesterol levels, were fed 100 g per day rice bran or oat bran for two 3-week periods in a crossover design ([Hegsted et al. 1993]). Prior to each bran phase, a control diet without bran was provided. Total cholesterol levels decreased when rice bran or oat bran was consumed, though neither of the brans had a significant effect on HDL- and VLDL- cholesterol or triglycerides ([Hegsted et al. 1993]). Another human study evaluated the effect of rice bran, oat bran and a rice starch placebo on moderately hypercholesterolemic, non-smoking, non-obese adults during a 6-week, randomised, double-blind trial ([Gerhardt and Gallo 1998]). The 23 males and 21 females were given 84 g of product per day to consume in addition to their regular diet. Significant total cholesterol reduction and improvement in the total cholesterol to HDL-cholesterol ratio in most of these individuals who consumed the bran was reported. Again, there was no significant difference between the effectiveness of the rice and oat bran ([Gerhardt and Gallo 1998]). Both these studies offer rice consumers an option that does not include changing their preferred staple.

Studies using hamsters and nonhuman primates have concluded that the rice bran oil fraction gives rice bran its cholesterol lowering properties ([Kahlon et al. 1991];[Nicolosi et al. 1991]). Studies using moderately-hypercholesteroleimic healthy humans (n = 26) in a parallel arm design and a randomised crossover design, have compared the effects of a diet including dietary fibre from rice bran or defatted rice bran, and dietary lipids from rice bran oil or another oil blend with a fatty acid composition similar to rice ([Most et al. 2005]). The study showed that defatted rice bran did not lower lipid levels, and consumption of the diet containing rice bran oil compared to the control diet resulted in lower total cholesterol levels ([Most et al. 2005]), suggesting that the oil in rice bran contains unique compound/s for lowering cholesterol. The fractions of primary focus for this capacity have been the tocotrienols, sterols, gamma-oryzanol and policosanols.

Less than 1% of all research published regarding the Vitamin E complex has focused on tocotrienols. Reviews indicate that most of the research on tocotrienols has focused on this fraction from palm oil or individual isomers, while only a limited focus has been placed on this fraction in rice bran ([Packer et al. 2001];[Rasool and Wong 2007];[Sen et al. 2007]). Research with cell cultures has shown that tocotrienols together and as individual isomers influence cholesterol synthesis by regulating the expression of 3-hydroxy-3-methylglutaryl-coenzyme A reductase, the rate-limiting enzyme in the cholesterol synthesis pathway ([Parker et al. 1993]). Interestingly, α-tocopherol has shown an opposite effect in hypercholesterolemic human subjects ([Qureshi et al. 2002]).

Conflicting conclusions have been reported from clinical trials that examined the effects of rice and palm tocotrienol rich fractions on cholesterol ([Packer et al. 2001];[Rasool and Wong 2007];[Sen et al. 2007]). The studies that reported an inhibitory effect of tocotrienols on total cholesterol levels used preparations with less than 20% tocopherols. The reverse was reported for studies that used preparations with a greater percentage of tocopherols. Rice bran reportedly contains approximately 25% tocopherols and 75% tocotrienols ([Bergman and Xu 2003]), so this could be one of the ways that rice bran lowers cholesterol. Potentially confounding aspects of the design of human trials have been that the relative amounts of the four tocotrienol isomers varied between these studies. Several of these studies, also, did not control the amount of dietary lipids and alcohol consumed by the subjects; both have been reported to modulate the effects of tocotrienols ([Qureshi et al. 1997]).

Policosanols are a mixture of primary long-chained alcohols. Sugarcane policosanols reduced plasma LDL cholesterol in several clinical trials of varying duration and at efficacious doses, ranging from 2 to 40 mg per day ([Chen et al. 2005];[Varady et al. 2003]). Whole-grain rice contains policosanols but its individual compounds are found in different ratios compared to the similar fraction in sugarcane. Rice policosanols (10 mg per day), when fed to hypercholesterolemic men and women in a randomised, double-blind, crossover, placebo-controlled trial (n = 70), reportedly lowered the subjects plasma total cholesterol, and increased levels of Apolipoprotein A-1, the major protein componenet of HDL ([Reiner et al. 2005]). However, a lack of cholesterol-lowering efficacy of sugarcane policosanols was reported from a study with a similar design to those reported above ([Berthold et al. 2006]). Thus, the association between policosanol consumption and plasma cholesterol reduction is unclear. To clarify this situation, future research will need to take into consideration that the specific compounds and amounts of each type in the policosanol fraction vary both between and within crops.

Numerous studies with rodents have reported that the gamma-oryzanol fraction from rice bran is able to lower serum cholesterol levels in animals fed different model hypercholesterolemic diets ([Cicero and Derosa 2005]). The mechanism of action appears to include increased faecal excretion of cholesterol and its metabolites ([Wilson et al. 2007]). These authors also reported that ferulic acid from rice bran showed anti-atherogenic properties, but through a different mechanism. The serum cholesterol lowering properties of gamma-oryzanol have not been confirmed in humans.

While it seems clear that there is an association between the consumption of whole grain rice and a lowering of cardiovascular risk factors, the mechanisms leading to this could be due to multiple compounds. In order for rice improvement programs to make use of these associations, there is a need for investment targeted specifically towards identifying the mechanism of risk reduction, understanding variability within rice for managing cardiovascular risk factors, and then using that information to develop phenotyping tools so that selection for heart-healthy varieties of rice is possible in the future.

#### Cancers and whole grain rice

A meta-analysis of prospective epidemiologic studies suggests that consumption of whole-grain products is inversely associated with the development of several forms of cancer ([Williams and Hord 2005]). However, only a limited number of human, animal and cell culture-based studies that specifically evaluate the association of whole grain rice, or it’s fractions, with cancer risks have been done, and many studies cannot be done in humans due to limitations in analytical capability. The studies indicate that whole grain rice contributes to mitigating cancers, tumour growth and proliferation by two different mechanisms. There is an increasing body of evidence that resistant starch (RS) in the whole grain provides one mechanism, and the second is through bioactive compounds that could be present in the bran layer of the rice. However, neither mechanism is well understood, but the results described below suggest that both mechanisms are worthy of pursuing further.

Colon cancers arise from benign neoplasms and evolve into adeno-carcinomas through an histological sequence beginning with either adenomas or hyperplastic polyps. A link between dietary fibre and reduced risk of colon cancer was first proposed several decades ago. That association remains controversial; however, confidence in a link between whole grain consumption and reduced risk of colorectal cancers is growing ([Annison and Topping 1994];[Bird et al. 2000];[Park et al. 2005];[Schatzkin et al. 2007];[Topping et al. 2003];[Topping 2007];[Topping and Clifton 2001]). Several mechanisms have been proposed to explain this possible association. Cereal bran may have a protective effect against colorectal cancer by altering the colonic bacteria profile via addition of fermentable carbohydrates, such as resistant starch (RS).

Recent evidence suggests that RS results in an increased production of short chain fatty acids (SCFA) such as butyrate in the colon ([Topping 2007]). These are considered to play a number of roles in bowel health, including recovery from chronic diarrhoea, lowering absorption of potential carcinogens, and repair of damaged DNA ([Topping 2007]). Studies using pigs found faecal SCFA levels higher when the pigs were fed whole grain rice relative to feeding with milled rice and bran. This was reportedly due to greater RS in the whole grain rice wherein the bran layer protected the starch from digestion, enabling the starch to ferment in the large intestine where it produced SCFA ([Bird et al. 2000]). Consistent with this, a systematic study of RS in a diverse set of rices showed that RS in unpolished cooked rice was 30% higher than in the polished cooked rice of the same variety ([Williams et al. 2005]), probably due to the bran layer preventing digestive enzymes from accessing the starch. RS can also be manipulated in polished grain. The starch branching enzyme IIb gene (*SBEIIb*) has a large effect on RS, and varieties that carry a mutation in this gene show elevated RS ([Butardo et al. 2011]), but when expression of that gene is completely silenced with specific transgenic techniques, the RS is significantly increased ([Butardo et al. 2011]).

Familial adenomatous polyposis is an hereditary condition that predisposes people to colon cancer. Apc(Min) mice carry a mutation in the same gene that causes familial adenomatous polyposis in humans. These mice develop large numbers of intestinal tumors at an early age and are thus used as a model for evaluating chemo-preventive interventions for humans with intestinal polyps. When rice bran was included in the diet in a cross-over experiment, the Apc(Min) mice showed a decrease of 51% in the number of intestinal adenomas ([Verschoyle et al. 2007]). The mechanism is unclear, but is likely to be due to compounds present in the bran.

Isoprenoids are known to possess potent anti-cancer activity ([Sen et al. 2007]), and whole grain rice contains several different types of these compounds. For example, γ-tocotrienol, the tocotrienol that rice has in the largest quantity, has been documented using tissue culture techniques to be the most potent anti-cancer Vitamin E isoform of all the isomers that occur in nature ([Sen et al. 2007]). Numerous studies documenting this observation have shown that tocotrienols and γ-tocotrienol in particular, target Nuclear Factor-κB (a transcription factor) which reduces inflammation and thus mediates the impact of carcinogens ([Ahn et al. 2007];[Nesaretnam and Meganathan 2011]). Work using mice not only supports the results found using cell lines, but when extrapolated to humans, indicates that an efficacious dose of tocotrienols could be consumed from the diet ([He et al. 1997]). Other isoprenoids in whole grain rice have been reported to interfere with the colony-forming ability of breast and colon cancer cells ([Hudson et al. 2000]). Eight phenolic compounds, protocatechuic acid ([Hudson et al. 2000]), ρ-coumaric acid ([Zhou et al. 2004]), caffeic acid ([Hudson et al. 2000]), ferulic acid ([Tian et al. 2004]), sinapic acid ([Hudson et al. 2000]), vanillic acid ([Zhou et al. 2004]), methoxycinnamic acid and tricin ([Hudson et al. 2000]), were identified in the extracts studied. Of these compounds, the flavonoid tricin has received the greatest research attention ([Zhou and Ibrahim 2010]) which has likely been due to its greater activity in interfering with cancer cells in tissue culture experiments at levels lower than the other phenolics studied, making it an easier compound to study than others.

**Figure 3:**
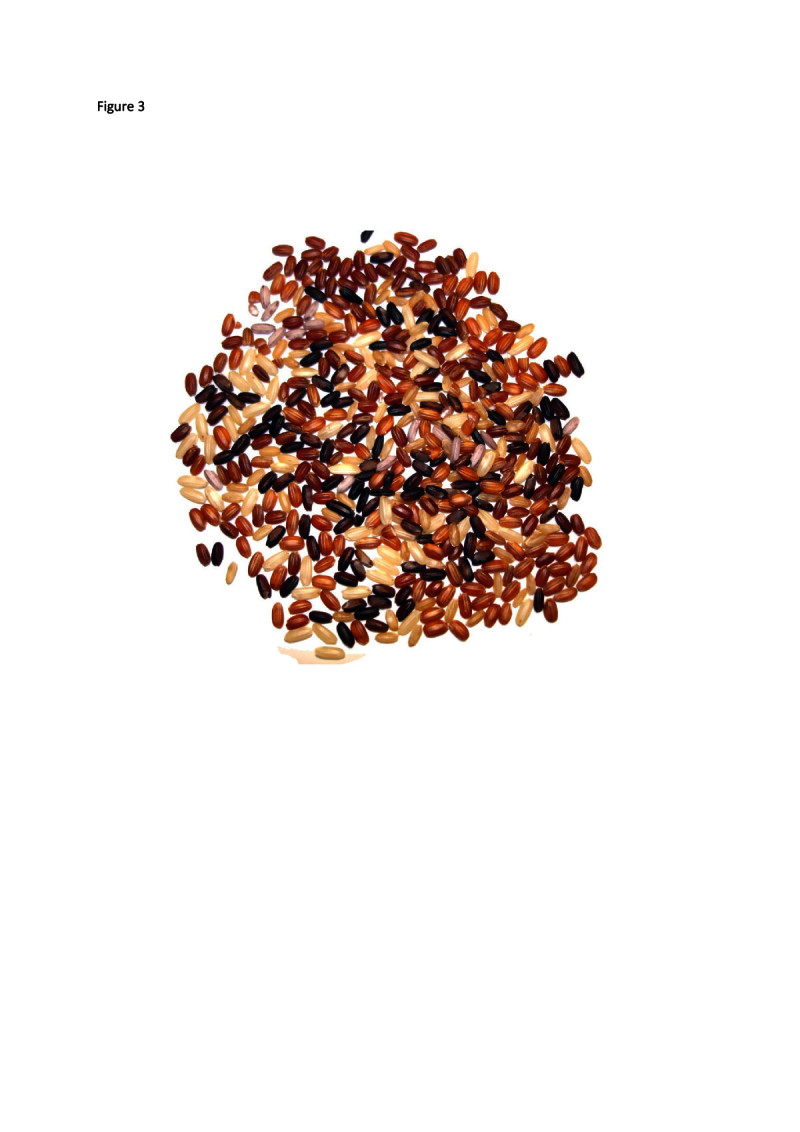
**Diversity in the colour of the bran of unpolished rice.**

During tumour metastasis, a critical early step is cell invasion of the basement membrane - a dense meshwork of collagen, glycoproteins, and proteoglycans which, under normal circumstances, prevents cells from moving away from their sites of origin. Cancer cells, however, secrete several different types of enzymes that digest the proteins in the basement membrane ([Fidler 2003]). When the membrane has been sufficiently weakened, the tumour is able to push through the membrane ([Liotta and Stetler-Stevenson 1991]), which allows cells to invade surrounding tissue. Another isoprenoid fraction from whole grain rice, specifically the anthocyanins cyanidin 3-glucoside and peonidin 3-glucoside were isolated and shown to inhibit the mobility and invasion ability of human hepatocellular carcinoma (SKHep-1) cells ([Chen et al. 2006]). This effect was associated with a reduced expression of several proteinases. In addition, these compounds were fed to mice after subcutaneous inoculation with SKHep-1 cells. Small solid tumours were observed following cell inoculation and a 1.9-fold reduction in tumour volume and a 1.7-fold reduction in tumour weight were reported after feeding the mice the anthocyanin fraction from whole grain rice ([Chen et al. 2006]). The anthocyanin, antioxidant and phenolic content ranges enormously in rice, and several studies associate it positively with coloured pericarps ([Goffman and Bergman 2004];[Han et al. 2004];[Oki et al. 2004];[Shen et al. 2009]). Figure3 shows the diversity in the colour of the rice pericarps.

The studies described above all indicate that whole grain rice plays a significant role in preventing cardiovascular risk factors, tumour growth and tumour proliferation, through both the mechanical protection of starch by the bran with the subsequent benefits of SCFA, and the range of bioactive compounds in bran. These data call for stronger links between rice researchers and the medical fraternity, significant investment in research to detect and identify the important grain constituents and to quantify the nutritional impact in humans, and techniques to deliver to rice improvement programs to enable selection on bran quality. The bran content of rice ranges from 5–8% ([Bergman and Chen 2007]), which could be an immediately selectable trait for rice varieties targeted to markets that consume either whole-grain rice, or different forms of it, such as pre-germinated rice, which is also consumed as whole-grain rice.

#### Type II diabetes mellitus and rice

Diabetes is a chronic evolving disease associated with a variety of micro- and macro-vascular complications. Although pharmacological therapies are effective, the diabetes prevention trials in Finland and the U.S. remind us that nutrition and lifestyle approaches can be more effective in delaying onset of the disease. In fact, these nutrition and lifestyle approaches to diabetes prevention and treatment should be given at least as much attention as drug therapies. The use of low glycaemic index (GI) foods should be considered as one of a number of tools available to manage, or prevent the onset of, Type II diabetes.

The GI of a food quantifies the rate of release of glucose into the blood in response to the carbohydrates consumed. The glycaemic load (GL) is the mathematical product of the GI and the amount of carbohydrate in the food. A recent meta-analysis of 37 prospective observational studies concluded that GI and GL are both strongly associated with Type II diabetes and its chronic diseases ([Barclay et al. 2008]). Another recent study confirms this ([Halton et al. 2008]), and a prospective cohort study and systematic review of six other cohort studies concluded that two-servings-per-day of a whole grain was associated with a 21% decrease in risk of type II diabetes ([Munter et al. 2007]). These studies consisted of 286,125 participants and 10,944 of these had Type II diabetes. A feeding trial of patients with Type II diabetes found that those on the diet of lower GI had a significant improvement in blood sugar status compared with those on the higher GI diet ([Nisak et al. 2010]), strongly suggesting that choice of carbohydrate will aid in the management of blood sugar status.

Rice has generally been considered to be a food of high GI ([Brand-Miller et al. 1992]). Several studies link the consumption of rice with increased risk of developing Type II diabetes ([Nanri et al. 2010];[Villegas et al. 2007]). Those studies were carried out in Japan and Shanghai respectively, and in both those places, the rices consumed are likely to be of high GI because they are low amylose varieties ([Sato et al. 2004];[Fitzgerald et al. 2011]). Both[Nanri et al.] (2010) and[Villegas et al.] (2007) report an association between diabetes and rice consumption, and the association was stronger in people with low physical activity coupled with high intake of rice. Another study based in Australia shows that the risk of developing Type II diabetes was highest in people who consume high levels of white bread together with low physical activity ([Hodge et al. 2004]). The GI of white bread is high ([Foster-Powell et al. 2002]). Together, the three studies indicate that consumption of carbohydrate of high GI is likely to increase risk of Type II diabetes, especially when coupled with low physical activity, irrespective of the grain delivering the carbohydrate. In areas where rice is the staple, correlations between consumption of high GI rice and Type II diabetes will be found, however where wheat is the staple, the correlations with Type II diabetes will be found for those who consume white bread. Furthermore, when the diversity of rice and rice products is explored, a significant range in GI has been demonstrated. For a set of 260 varieties of polished rice, GI ranged from 52 – 92 ([Fitzgerald et al. 2011]). Rice products, such as parboiled rice and rice vermicelli, have also been shown to give a GI of at least 10 units below the GI of the white rice of the same variety ([Ranawana et al. 2009];[Sato et al. 2010]). These studies suggest that particular varieties of rice and rice products can be chosen to manage Type II diabetes, or possibly prevent the onset of the condition.

It is currently unclear whether whole-grain rice (unpolished) offers any advantage over polished rice for the management of blood glucose. A prospective study in the US reported that consumption of polished rice led to a greater likelihood of developing Type II diabetes than consumption of whole-grain rice ([Sun et al. 2010]). However,[Sun et al. (2010]) compared the average GI of brown and white rice published by[Foster-Powell et al. (2002]), but only three varieties were tested as both brown and white rice. Comparing the GI values of the brown and white rice of the three varieties, Doongara, Amaroo and Pelde, showed no difference in GI for the brown and white forms of Doongara and Amaroo ([Foster-Powell et al. 2002]). Furthermore, other differences between the levels of physical activity and other dietary choices of the brown and white rice consumers studied by[Sun et al. (2010]) could easily contribute to the difference in the incidence of Type II diabetes. Two earlier studies that compared the GI of different varieties of whole-grain (brown) rice with the polished rice of the same variety gave conflicting results. The first study measured GI in eight healthy volunteers who consumed whole-grain and polished rice in a randomised experiment, and no difference in blood glucose response was found between the pairs of whole-grain and polished for the three varieties ([Brand-Miller et al. 1992]). The second study found significantly lower blood glucose responses when participants consumed the whole-grain rice compared to the polished rice of the same variety ([Panlasigui and Thompson 2006]). These differences could be due to varietal differences, differences in cooking, differences in measuring GI, or other factors of diet and lifestyle. It is therefore not yet possible to conclude if the consumption of whole-grain rice gives any advantage over polished rice for the management of blood glucose status.

The Nurses' Health Study and the Harvard School of Public Health indicate that higher intakes of Mg may reduce the risk of developing Type II diabetes ([Lopez-Ridaura et al. 2004]). Research has shown that low levels of Mg may impair insulin sensitivity or function. Consuming adequate levels of Mg may help insulin function properly in the body, which may assist in preventing Type II diabetes. Table1 shows a significant amount of Mg is found in the whole-grain, but polishing to white rice removes 86% of it ([Hansen et al. In Press]).

The diversity of compounds found in the bran, metabolites and minerals in the grain, structures of starch, non-starch polysaccharides, and different cooking and processing methods are all likely to have some impact on the digestibility of rice. Given that rice consuming countries face a grand challenge with skyrocketing rates of Type II diabetes, it is essential that we understand a lot more about the digestibility of rice and the importance and roles of other bioactive compounds from rice and bran. Investment is needed to enable science to identify and validate important compounds, and deliver this knowledge, as phenotyping tools, to rice improvement programs.

#### Consumption of whole grain rice

Unpolished rice is not widely consumed world-wide, and is most likely to be consumed in Western countries by health-conscious consumers. It can be found in the markets in most Asian countries, but the belief is that the unpolished rice is for the elderly, and anecdotally it is said to provide nutritional and metabolic benefits. However, there are two methods of processing rice that are likely to provide some of the nutritional value of whole-grain to consumers. The first is parboiling of the paddy before it is polished to white rice. The second is the relatively new technique of pregerminating the brown rice grain prior to cooking the brown rice, which alters the biochemical profile of the bran and improves the cooking and sensory properties of brown rice.

Parboiling involves three basic processes prior to dehulling and polishing: soaking (or steeping), steaming/boiling, and drying ([Chukwu 1999]). Parboiling is practiced in India, Pakistan, Sri Lanka, Bangladesh, West Africa, the Americas and Europe, and the methods used are all slightly different. After parboiling, the rice is polished before it is consumed, and the polished parboiled rice is considered to be of superior nutritional value compared to polished rice that has not been parboiled ([Amato et al. 2002];[Pedersen et al. 1989]). The nutritional advantage of parboiled polished rice is thought to be due to the leaching of minerals and water-soluble vitamins from the bran layers into the endosperm during the parboiling process ([Amato et al. 2002];[Juliano 1985];[Nunes et al. 1991]). However, not all migrating minerals are recovered in the endosperm. Comparisons between the mineral content of brown (not parboiled) and parboiled brown rice of the same variety show slight variations for the concentration of Fe, Zn and Ca, suggesting that a proportion of these minerals was lost during the parboiling process ([Heinemann et al. 2005]). Two studies have also shown a slight decrease in carotenoid content of parboiled rice (0.003 mg to 0.001 mg/100 g) ([Gariboldi 1973];[Otegbayo et al. 2001]), again, presumably due to leaching. Parboiling leads to the migration of thiamine, riboflavin and bran pigments, such as bioactive anthocyanins, into the grain ([Lamberts et al. 2006];[Manful et al. 2007]). However, there is no systematic study quantifying (i) leaching from the bran into the endosperm, or (ii) the nutritional value of polished parboiled rice due to different parboiling treatments, and no information can be found about the leaching patterns of the classes of bioactive compounds discussed above.

Pregermination is another processing method that could enable consumers to avail of the nutritional value of bran. In this process, the brown rice is soaked at 37°C for 24 h to initiate the germination process ([Finney 1983];[Sakamoto et al. 2007]). The rice is then dried, and vacuum-sealed, and it is not polished prior to consumption. Pregermination leads to extensive biochemical changes in the pregerminated rice compared with the ungerminated whole-grain ([Kayahara and Kikuichi 2000];[Sakamoto et al. 2007]). These changes lead to significant increases in bioactive compounds such as γ-aminobutyric acid (GABA), dietary fibre, inositols, ferulic acid, phytic acid, tocotrienols, Mg, K, Zn, γ-oryzanol, and prolylendopeptidase inhibitor ([Kayahara and Kikuichi 2000]). Total phenolics increased to a maximum level after a germination time of 24 hours while GABA, which was not present before germination, developed in significant quantities upon pre-germination ([Hirunpong and Tungjaroenchai 2008]). Novel acylated steryl glucosides appeared at the same level of bioactivity as found in soybean after pre-germination ([Usuki et al. 2007]). In countries of South-East and North Asia, pregerminated rice is marketed with the nutritional label of GABA rice ([Panchan and Naivikul 2009]).

Germination provides further nutritional value by reducing anti-nutritional factors in whole-grain rice, such as phytate ([Liang et al. 2008];[Shallan et al. 2010]). Phytate inhibits the bioavailability of minerals by forming stable, indigestible complexes ([Ma et al. 2005];[Welch and Graham 2004]). The process of germination activates the production of phytase, which catalyses the hydrolysis of phytate ([Sung et al. 2005]). Moreover, the sensory properties of pregerminated rice are considered to be superior to those of brown rice ([Fujino and Kuwata 2004]), and this, together with the nutritional enhancement, could increase the acceptability of pregerminated rice amongst consumers of polished rice.

Exploring genetic variability for the response to pregermination could lead to a wealth of new information about the nutritional potential of rice. Partnerships with the medical profession could enable nutritional impact to be quantified, which could then flow through to rice improvement programs, to improve the nutritional value of rice by maximising the potential of the raw material, and of the process.

### New technologies for selecting tools for nutritional quality

With the importance and relevance of nutritional components being clear, there is an increasing demand for more detailed information on the molecular mechanisms behind the biochemical content of the rice grain, both before harvest and after polishing. New high throughput phenotyping platforms such as metabolomics, giving unprecedented insights into grain composition, and HTP genotyping platforms such as Next Generation Sequencing, RNAseq and SNP arrays, which reveal the genetics behind varietal differences, have huge potential.

### Metabolomics as a new phenotyping tool for nutritional compounds

Metabolomics approaches for plants have now been around for little more than 10 years ([Fiehn et al. 2000]), and give us the capacity to study the biochemical composition of plant materials in an untargeted manner ([Hall 2006];[Hall 2011]). The biochemical profiles obtained, using a range of now standard platforms, cover most of the main groups of nutritionally-relevant small molecules. Large (polymer) molecules are not included, which makes metabolomics an excellent complement to other approaches targeting key nutrient groups, such as starch and proteins ([Fernie and Schauer 2009];[Hall 2006]). Our knowledge of the plant metabolome is still limited, as was demonstrated recently using rice as an example to show, that even after a detailed (literature) analysis of all available information on this crop’s metabolites, we must still only have visualised just a small fraction ([Kind et al. 2009]). Metabolomics is helping to increase this coverage, but much work is still needed if we are to link e.g. key nutrient traits with mixtures of known metabolites ([Fernie and Keurentjes 2011];[Stewart et al. 2011]).

For rice, only a few true metabolomics studies have been published to date (see ([Hall et al. 2008];[Oikawa et al. 2008];[Tarpley and Roessner 2007])). Nevertheless, the potential of the technology has clearly been recognised, particularly in areas such as stress tolerance ([Ahuja et al. 2010];[Tarpley and Roessner 2007]), grain development, grain quality, and nutritional value ([Fitzgerald et al. 2009];[Yamakawa and Hakata 2010]). While early rice metabolomics was often performed on leaf material (Capillary Electrophoresis MS, ([Sato et al. 2008];[Sato et al. 2004]), later, additional approaches have been used to analyse rice grains (GC-MS on transgenic rice, ([Zhou et al. 2008]); 1D and 2D GC-MS on brown rice, ([Kusano et al. 2011]); HSSE/GC-MS on rice grains, ([Grimm et al. 2011])). Only one metabolomics study has so far been published on rice bran, but specifically in the context of its use as a suitable starting material for fermentation to produce health-promoting phytochemicals ([Ryan et al. 2011]). Methods for the untargeted analysis of rice grain volatile compounds, which are important in determining key phenotypic traits in fragrant rices such as Basmati and Jasmine types, have also recently been published ([Boualaphanh et al. 2011a];[Calingacion et al. 2011];[Verhoeven et al. 2011]). GC-MS results have revealed that rice fragrance (measured from extracts or directly from head space) is created from a rich mix of a wide range of chemically-diverse compounds ([Bryant and McClung 2011];[Calingacion et al. 2011];[Champagne et al. 2008]). Many of these can be associated with positive fragrance traits, but others can be linked to off-flavour/negative traits. Advanced knowledge of both is essential when we wish to design breeding programmes with increased capability to select for nutritional quality. Using LC-MS, ([Heuberger et al. 2010]) also revealed a vast richness in the non-volatile metabolites, many of which could be associated with nutritional value or potential health benefits.

In the most extensive integrated approach to date,[Calingacion et al., (2011]) have used five complementary metabolomics platforms (NMR, LC-MS, GC-MS, GC-TOF-MS and ICP-MS) to gain a broad insight into minerals and metabolites in polished rice grains. Three contrasting genotypes were used in a proof of concept experiment, and it was demonstrated that all platforms could readily distinguish each genotype. This indicates that irrespective of analytical platform or group of metabolites studied, chemical diversity is sufficient to enable genotype-specific profiles to be identified. This individuality in diversity is presumably also the fundamental basis of the high discriminatory potential of the human palette to distinguish rice varieties after cooking, even when the grains come from genetically closely related varieties ([Calingacion et al. 2011];[Champagne et al. 2010]). Of course, metabolic complexity is, to a large extent, the reflection of complexity resulting from genetic polymorphism. Our growing potential to exploit the sequence of the rice genome, in combination with e.g. full genome transcriptomics, comprehensive SNP genotyping and genome-wide association studies using multi-platform metabolomics studies ([Calingacion et al. 2011]), is predicted to enable us to make unprecedented steps in our quest to understand better the molecular basis of rice metabolite profiles, and design tailor-made nutrition-directed breeding strategies for specific rice varieties, whole-grain and polished, to meet future nutritional needs.

### New genotyping tools for defining the genetics of nutritional quality

Over the last ten years, genotyping tools have advanced immeasurably. Genome-wide genotyping has become almost routine, and now the current advances are increasing the resolution and application of the technology to rice exponentially ([McCouch et al. 2002]). Now, single nucleotide polymorphisms (SNPs) are replacing most genotyping techniques, and with new SNP techniques, DNA from a single variety can be screened at many loci in a single pass ([Boualaphanh et al. 2011b];[McCouch et al. 2010]).

Routine genotyping at 384 SNP loci is currently possible for rice, for both *indica* and *japonica* subspecies ([Boualaphanh et al. 2011b];[Wright et al. 2010]). However, just as genotyping at 384 loci has become routine, the number of loci on newly developed chips has risen to 44000, and is soon to reach 1, 000,000 loci ([Tung et al. 2011]). As this rich collection of SNPs are discovered, the genotyping can be used to develop mapping populations rapidly ([Boualaphanh et al. 2011b]), or used to associate with phenotype data ([Calingacion et al. 2011]), or a particular set of SNPs can be selected and used for specific genotyping in a breeding program ([Chen et al. 2011]).

Once phenotyping tools can identify compounds and structures that offer nutritional benefit, the new genotyping tools offer the opportunity for rapid discovery of the genes responsible for the phenotype. Such genetic knowledge can then be delivered to breeding programs to enable genetic selection for compounds, structures and traits that offer nutritional benefit.

### Consumer acceptance of nutritionally enhanced rice

Consumers have eaten particular varieties for many generations in the belief that they give nutritional value. For example, rice in India with red bran is marketed and prescribed by Ayurvedic practioners for its health benefits such as controlling hypertension and diabetes ([Ahuja et al. 2008]). In Laos, specific varieties are consumed by post-partum women ([Bounphanousay 2007]). Belief in these effects on health has been upheld for many generations, but the mechanisms through which they act are not clear. Any rice that is sold unpolished in the U.S. can legally be marketed with the following FDA-approved health claim: “Diets rich in whole-grain foods and other plant foods and low in total fat, saturated fat, and cholesterol may reduce the risk of heart disease and some cancers” ([FDA 1999]). However, labels are not specific, mechanisms are not well understood, impact on health is not quantified, and consumption of whole-grain rice is very low in developing countries. The purpose of the present review is to create awareness of the nutritional potential of rice; identify current progress with developing varieties for specific issues of malnutrition and chronic disease, through the understanding the constituents of the rice grain, and bringing new science to bear; and identifying new opportunities to tap into the diversity of rice and identify or develop additional nutritionally enhanced varieties. However, there are a number of issues to consider when marketing such varieties to policy makers, farmers and most importantly, consumers.

Some countries have very strict food labeling regulations, such as in Japan, while others have relatively lax laws, as in China ([Hawkes 2004]). In regions of the world with strict regulations, marketing rice as possessing a certain level of a nutrient will require that the levels are documented through laboratory testing. Labeling rice with a health claim will be particularly challenging since countries have diverse requirements for making such claims, and consumers believe claims to varying degrees ([Shimizu 2011]). Furthermore, in most regions of the world, cultivars with an enhanced nutrition profiles, such as those with elevated Zn, will need to be segregated from those without the trait so the impact is not diluted. Segregation of particular cultivars is known as identity preservation (IP) across the crop world. IP requires cultivars to be harvested, stored, and marketed separately ([Sundstrom et al. 2002]). These systems specify that particular standards be upheld, records kept, and testing be performed. In other situations IP is used to enable consumers to choose products without a particular trait, as in the case of genetically modified (GM) crops. The European Union, Japan, Australia and other regions of the world have laws requiring the labeling of GM foods ([Huffman 2004]). Thus, IP will likely be required for a nutritionally enhanced rice to be sold whether it is created using traditional plant breeding techniques, mutation breeding, or genetic engineering.

Bringing a nutritionally enhanced rice to a consumer or a patient has its own set of obstacles. The first of these is that it is necessary to meet some of the nutritional challenges using the tools of transgenic technology. Golden rice was developed with genes from another species (maize) ([Grusak 2005]), but the high Fe rice that best meets nutritional targets was developed using a transgenic technique to enable a gene found in all rice varieties, that usually expresses in the roots and shoots, to express in the grain as well ([Johnson et al. 2011]). These are different degrees of genetic modification, but will that influence consumer choice? The development of transgenic food products has been highly controversial, in spite of the fact that there was no other way to create grains of rice with nutritionally useful levels of iron or rice grains containing β-carotene. Generally consumer skepticism is focused around questions of unknown environmental and health consequences of growing or consuming the transgenic products ([Curtis et al. 2004]). The two transgenic products described here have been developed with the specific humanitarian objective of improving the health of the poorest people. However, consumers and policy makers have every right to ask questions, and should do so. As more trials are undertaken in different locations and in target populations, science will begin to provide the answers to the critical questions, which then will enable policy makers and consumers to make informed decisions.

A second obstacle to consumer acceptance concerns the capacity of science to quantify the nutritional impact of the nutritionally valuable rices, especially with regard to chronic diseases, and to communicate that effectively to the medical community. At present, studies investigating the impact of nutritionally enhanced rice in preventing chronic disease, or developing therapeutic strategies for controlling these, are lacking, and specific dietary recommendations are not focal points in the management of chronic diseases by the medical community.

Presently, the medical community primarily practices evidence-based medicine, which aims to apply the best available evidence gained from the scientific method to clinical decision-making. Quantifiable, evidence-based data based on extensive research would be invaluable for encouraging more medical practitioners to combine dietary recommendations with medicinal treatment as their primary strategy in managing patients with chronic diseases. However current limitations to this are the identification of grain constituents with health benefits, and the existence of rapid and accurate methods that enable data to be gathered to quantify medical impact. Techniques for testing nutritional claims from rice research would provide the conduit for collaboration between the medical community and rice scientists to enable rice varieties to be developed to provide solutions to chronic diseases.

## Conclusions

Prioritising investment into identifying the biological causes underlying associations between rice consumption and decreased risk factors for chronic diseases would have significant and long-term impact on global nutritional challenges that have their greatest effect in developing countries. Technologies are advancing at rates rapid enough to make scientific progress in these areas, and doing so would lead to both nutritional and economic benefit in every rice-consuming country. The outputs from such research programs must be integrated with other efforts aiming to deliver climate-ready varieties that resist the challenges of the changing environment, and varieties that are acceptable to consumers. Rice that does not meet the needs of consumers in terms of physical and sensory properties will not be successful in the marketplace, but once research programs are resourced to understand the linkages between chemicals in rice and human health, and are able to deliver solid nutritional information to the medical community, consumers might be able to be persuaded to increase their consumption of wholegrain or pregerminated rice.

## Authors’ original submitted files for images

Below are the links to the authors’ original submitted files for images. Authors’ original file for figure 1 Authors’ original file for figure 2 Authors’ original file for figure 3

## References

[CR1] Ahn KS, Sethi G, Krishnan K, Aggarwal B (2007). Gamma-tocotrienol inhibits nuclear factor-kappaB signaling pathway through inhibition of receptor-interacting protein and TAK1 leading to suppression of antiapoptotic gene products and potentiation of apoptosis. J Biol Chem.

[CR2] Ahuja U, Ahuja SC, Thakrar R, Singh RK (2008). Rice – A Nutraceutical. Asian Agri-History.

[CR3] Ahuja I, de Vos CHR, Bones A, Hall RD (2010). Plant molecular stress programs face climate change. Trends Plant Sci.

[CR4] Akihisa T, Yasukawa K, Yamaura M, Ukiya M, Kimura Y, Shimizu N, Arai K (2000). Triterpene alcohol and sterol ferulates from rice bran and their anti-inflammatory effects. J Agric Food Chem.

[CR5] Amato GW, Carvalho JLV, Silveira (2002). parboiled rice fs: clean, noble product.

[CR6] Amaya D, Viloria H, Ortega P, Gómez G, Urrieta J, Lobo P, Estévez J (2002). Vitamin A deficiency and the anthropometric nutritional status of urban and rural marginalized children in the state of Zulia, Venezuela. Investigation Clinic.

[CR7] Amissah JGN, Ellis WO, Oduro I, Manful JT (2003). Nutrient composition of bran from new rice varieties under study in Ghana. Food Control.

[CR8] Annison G, David L, Topping (1994). Nutritional role of resistant starch: chemical stucture vs. physiological function. Annu Rev Nutr.

[CR9] Arsenault JE, Yakes EA, Hossain MB, Munirul Islam M, Ahmed T, Hotz C, Lewis B, Rahman AS, Jamil KM, Brown KH (2010). The current high prevalence of dietary zinc inadequacy among children and women of rural Bangladesh could be substantially ameliorated by zinc biofortication of rice. J Nutr.

[CR10] Astrup A, Dyerberg J, Selleck M, Stender S (2008). Nutrition transition and its relationship to the development of obesity and related chronic diseases. Obes Rev.

[CR11] Barclay AW, Petocz P, McMillan-Price J, Flood VM, Prvan T, Mitchell P, Brand-Miller JC (2008). Glycemic index, glycemic load, and chronic disease risk–a meta-analysis of observational studies. Am J Clin Nutr.

[CR12] Bergman C, Chen M (2007). Not all rice bran is created equal. beneath the hull: exploiting the health-beneficial properties of the rice grain.

[CR13] Bergman C, Xu Z (2003). Genotype and environment effects on tocopherol, tocotrienol, and gamma-oryzanol contents of Southern US rice. Cereal Chemistry.

[CR14] Berthold HK, Unverdorben S, Degenhardt R, Bulitta M (2006). GouniBerthold. 2006. Effect of policosanol on lipid levels among patients with hypercholesterolemia or combined hyperlipidemia: a randomized controlled.trial. J Am Med Assoc.

[CR15] Beyer P, Al-Babili S, Ye X, Lucca P, Schaub P, Welsch R, Potrykus I (2002). Golden Rice: Introducing the β-carotene biosynthesis pathway into rice endosperm by genetic engineering to defeat Vitamin A deficiency. J Nutr.

[CR16] Bhutta ZA, Haider BA (2009). Prenatal micronutrient supplementation: Are we there yet?. Can Med Assoc J.

[CR17] Bird AR, Hayakawa T, Marsono Y, Gooden JM, Record IR, Correll RL, Topping DL (2000). Coarse brown rice increases fecal and large bowel short-chain fatty acids and starch but lowers calcium in the large bowel of pigs. J Nutr.

[CR18] Black RE, Allen LH, Bhutta ZA, Caulfield LE, de Onis M, Ezzati M, Mathers C, Rivera J (2008). Maternal and child undernutrition: global and regional exposures and health consequences. Lancet.

[CR19] Boualaphanh C, Calingacion M, Jothityangkoon D, Sanitchon J, Cuevas RM, Fitzgerald M (2011). Yield and quality of non-aromatic and aromatic Lao rice in response to nitrogen fertilizer. Science Asia.

[CR20] Boualaphanh C, Daygon D, Calingacion M, Jirawat, Jongithoon D, Mumm R, Hall R, Fitzgerald M (2011). Use of new generation SNP genotyping for rapid development of near-isogenic lines in rice. Crop Science.

[CR21] Bouis H, Hunt J (1999). Linking food and nutrition security: past lessons and future opportunities. Asian Developement Review.

[CR22] Bounphanousay C (2007). Use of phenotypic characters and DNA profiling for classification of the genetic diversity in black glutinous rice of the Lao PDR. Agriculture.

[CR23] Brackmann C, Bengtsson A, Alminger ML, Svanberg U, Enejder A (2011). Visualization of β-carotene and starch granules in plant cells using CARS and SHG microscopy. Journal of Raman Spectroscopy.

[CR24] Brand-Miller JC, Pang E, Bramall L (1992). Rice: a high or low glycemic index food?. Am J Clin Nutr.

[CR25] Bryant RJ, McClung AM (2011). Volatile profiles of aromatic and non-aromatic rice cultivars using SPME/GC-MS. Food Chem.

[CR26] Butardo VM, Fitzgerald MA, Bird AR, Gidley MJ, Flanagan BM, Larroque O, Resurreccion AP, Laidlaw HK, Jobling SA, Morell MK, Rahman S (2011). Impact of Down-regulation of Starch Branching Enzyme IIb in Rice by Artificial microRNA and Hairpin-RNA-Mediated RNA Silencing. J Exp Bot.

[CR27] Calingacion M, Boualaphan C, Daygon D, Anacleto R, Biais B, Deborde C, Maucourt M, Moing A, Mumm R, Ric De Vos CH, Erban A, Hansen T, Laursen K, Shoerring J, Kopka J, Hall R, Fitzgerald M (2011). A metabolomics approach to identify new traits of rice quality in traditional and improved varieties of Laos. Metabolomics.

[CR28] Champagne ET, Bett-Garber KL, Thomson JL, Shih FF, Lea J, Daigle K (2008). Impact of presoaking on flavor of cooked rice. Cereal Chemistry.

[CR29] Champagne ET, Bett-Garber KL, Fitzgerald MA, Grimm C, Lea J, Ohtsubo K, Jongdee S, Xie L, Bassinello P, Resurreccion AP, Ahmad R, Habibi F, Reinke R (2010). Important sensory properties differentiating premium rice varieties. Rice.

[CR30] Chen JT, Wesley R, Shamburek RD, Pucino F, Csako G (2005). Meta-analysis of natural therapies for hyperlipidemia: plant sterols and stanols versus policosanol. Pharmacotherapy.

[CR31] Chen PN, Kuo WH, Chiang CL, Chiou HL, Hsieh YS, Chu SC (2006). Black rice anthocyanins inhibit cancer cells invasion via repressions of MMPs and u-PA expression. Chem Biol Interact.

[CR32] Chen H, He H, Zou Y, Chen W, Renbo Y, Liu X, Yang Y, Gao Y-M, Jian-Long X, Fan L-M, Li Y, Li Z-K, Deng X (2011). Development and application of a set of breeder-friendly SNP markers for genetic analyses and molecular breeding of rice (<i > Oryza sativa</i > L.). TAG Theoretical and Applied Genetics.

[CR33] Chukwu O (1999). Parboiling of rice paddy with heated pebbles. Journal of Science, Technology and Mathematics Education.

[CR34] Cicero AFG, Derosa G (2005). Rice bran and its main components: potencial role in the management of coronary risk factors. Current Topics in Nutraceutical Research.

[CR35] Curtis KR, McCluskey JJ, Wahl TI (2004). Consumer acceptance of genetically modified food products in the developing world. The Journal of Agrobiotechnology and Economics.

[CR36] Davy BM, Melby CL, Beske SD, Ho RC, Davrath LR, Davy KP (2002). Oat consumption does not affect resting casual and ambulatory 24-h arterial blood pressure in men with high-normal blood pressure to stage I hypertension. J Nutr.

[CR37] Eggum BO (1979). The nutritional value of rice in comparison with other cereals.

[CR38] FDA (1999). Health claim notification for whole grain foods.

[CR39] Fernie AR, Schauer N (2009). Metabolomics-assisted breeding: a viable option for crop improvement?. Trends Genet.

[CR40] Fernie, Alisdair R, Joost J, Keurentjes B (2011). Genetics, genomics and metabolomics. In Annual Plant Reviews.

[CR41] Fidler IJ (2003). The pathogenesis of cancer metastasis: the 'seed and soil' hypothesis revisited. Nat Rev Cancer.

[CR42] Fiehn O, Kopka J, Dörmann P, Altmann T, Trethewey RN, Willmitzer L (2000). Metabolite profiling for plant functional genomics. Nat Biotechnol.

[CR43] Finney PL (1983). Effect of germination on cereal and legume nutrient changes and food and feed value: a comprehensive review.

[CR44] Fitzgerald M (2007). Screening the International Rice Genebank Collection for Variation in Carotenoid Content Harvestplus and IRRI.

[CR45] Fitzgerald MA, McCouch SR, Hall RD (2009). More than just a grain of rice, the global quest for quality. Trends Plant Sci.

[CR46] Fitzgerald M, Concepcion J, Rahman S, Resurreccion A, Bird AR, Morell MK (2011). Identification of a major genetic determinant of glycaemic index in rice. Rice.

[CR47] Flight I, Clifton P (2006). Cereal grains and legumes in the prevention of coronary heart disease and stroke: A review of the literature. Eur J Clin Nutr.

[CR48] Foster-Powell K, Holt SHA, Brand-Miller JC (2002). International table of glycemic index and glycemic load values: 2002. Am J Clin Nutr.

[CR49] Fujino Y, Kuwata J (2004). Food functionality of sprout rice grain, super rice.

[CR50] Gariboldi F (1973). Rice testing methods and equipment.

[CR51] Gerhardt AL, Gallo NB (1998). Full-fat rice bran and oat bran similarly reduce hypercholesterolemia in humans. J Nutr.

[CR52] Ghaffar A, Reddy KS, Singhi M (2004). Burden of non-communicable diseases in South Asia. Br Med J.

[CR53] Goffman FD, Bergman C (2002). Relationship between hydrolytic rancidity, oil concentration and esterase activity in rice bran. Cereal Chemistry.

[CR54] Goffman FD, Bergman CJ (2004). Rice kernel phenolic content and its relationship with antiradical efficiency. J Sci Food Agric.

[CR55] Goffman F, Pinson S, Bergman C (2003). Genetic diversity for lipid content and fatty acid profile in rice bran. J Am Oil Chem Soc.

[CR56] Gregorio GB (2002). Progress in breeding for trace minerals in staple crops. J Nutr.

[CR57] Grimm CG, Champagne ET, Lloyd SW, Easson M, Condon B, McClung AM (2011). Analysis of 2-Acetyl-1-pyrolline in rice by HSSE/GC/MS. Cereal Chemistry.

[CR58] Grusak MA (2005). Golden Rice gets a boost from maize. Nat Biotech.

[CR59] Haas JD, Beard JL, Murray-Kolb LE, del Mundo AM, Felix A, Gregorio GB (2005). Iron-Biofortified Rice Improves the Iron Stores of Nonanemic Filipino Women. J Nutr.

[CR60] Hall RD (2006). Plant metabolomics: from holistic hope, to hype, to hot topic. New Phytol.

[CR61] Hall RD, Hall RD (2011). Plant Metabolomics in a nutshell: potential and future challenges. Biology of Plant Metabolomics.

[CR62] Hall RD, Brouwer ID, Fitzgerald MA (2008). Plant metabolomics and its potential application for human nutrition. Physiol Plant.

[CR63] Hallfrisch J, Daniel J, Scholfield, Kay M, Behall (2003). Blood pressure reduced by whole grain diet containing barley or whole wheat and brown rice in moderately hypercholesterolemic men. Nutr Res.

[CR64] Halton TL, Liu S, Manson JoAnn E, Frank BHu (2008). Low-carbohydrate-diet score and risk of type 2 diabetes in women. Am J Clin Nutr.

[CR65] Han SJ, Ryu S, Noh KSS (2004). A new 2-arylbenzofuran with antioxidant activity from the black colored rice (Oryza sativa L.) bran. Chem Pharm Bull.

[CR66] Hansen TH, Lombib E, Fitzgerald MA, Husted S, Boualaphanh C, Resurreccion A, Paterson D, Schjoerring JK (submitted). Losses of essential mineral nutrients by polishing of rice differ among genotypes due to contrasting grain hardness and mineral distribution. Cerea Chemistry.

[CR67] Hawkes C (2004). Nutrition Labels and Health Claims: The Global Regulatory Environment.

[CR68] He L, Mo H, Hadisusilo S, Asaf A, Qureshi, Elson CE (1997). Isoprenoids suppress the growth of murine B16 melanomas In vitro and In vivo. J Nutr.

[CR69] Hegsted M, Windhauser MM, Kay Morris S, Lestera SB (1993). Stabilized rice bran and oat bran lower cholesterol in humans. Nutr Res.

[CR70] Heinemann RJB, Fagundes PL, Pinto EA, Penteado MVC, Lanfer-Marque UM (2005). Comparative study of nutrient composition of commercial brown, parboiled and milled rice from Brazil. Journal of Food Composition and Analysis.

[CR71] Heuberger AL, Lewis MR, Chen M-H, Brick MA, Leach JE, Ryan EP (2010). Metabolomic and functional genomic analyses reveal varietal differences in bioactive compounds of cooked rice. PLoS One.

[CR72] Hirunpong P, Tungjaroenchai W (2008). Effect of Germination on Contents of Bioactive Components in Germinated Brown Rice of Three Rice Cultivars.

[CR73] Hodge AM, English DR, O’Dea K, Giles GG (2004). Glycemic index and dietary fiber and the risk of type 2 diabetes. Diabetes Care.

[CR74] Hossain P, Kawar B, El NM (2007). Obesity and diabetes in the developing world – a growing challenge. N Engl J Med.

[CR75] Hotz C, Brown KH (2004). Assessment of the risk of zinc deficiency in populations and options for its control. Food Nutr Bull.

[CR76] Hudson E, Dinh P, Kokubun T, Simmonds M, Gescher A (2000). Characterization of potentially chemopreventive phenols in extracts of brown rice that inhibit the growth of human breast and colon cancer cells. Cancer Epidemiol Biomarkers Prev.

[CR77] Huffman WE (2004). Production, identity preservation, and labeling in a marketplace with genetically modified and non-genetically modified foods. Plant Physiol.

[CR78] Hunt JR, Johnson LK, Juliano BO (2002). Bioavailability of zinc from cooked Philippine milled, undermilled, and brown rice, as assessed in rats using growth, bone zinc, and zinc-65 retention. J Agric Food Chem.

[CR79] Jacobs DR, Andersen LF, Blomhoff R (2007). Whole-grain consumption is associated with a reduced risk of noncardiovascular, noncancer death attributed to inflammatory diseases in the Iowa women's health study. Am J Clin Nutr.

[CR80] James WPT (2008). The fundamental drivers of the obesity epidemic. Obes Rev.

[CR81] Jamison DT, Mosley HW, Measham AR, Bobadilla JL (1993). Disease control priorities in developing countries.

[CR82] Jiang SL, Wu JG, Feng Y, Yang XE, Shi CH (2007). Correlation analysis of mineral element contents and quality traits in milled rice (Oryza sativa L.). J Agric Food Chem.

[CR83] Johnson AT, Kyriacou B, Callahan DL, Carruthers L, Stangoulis J, Lombi E, Tester M (2011). Constitutive overexpression of the OsNAS Gene family reveals single-gene strategies for effective iron- and zinc-biofortification of rice endosperm. PLoS One.

[CR84] Juliano BO (1985). Rice properties and processing. Food Reviews International.

[CR85] Kahlon TS, Chow FI, Sayre RN, Betschart AA (1991). Cholesterol-lowering in hamsters fed rice bran at various levels, defatted rice bran and rice bran oil.

[CR86] Kayahara H, Kikuichi T (2000). Flavor.

[CR87] Kind T, Scholz M, Fiehn O (2009). How large is the metabolome? A critical analysis of data exchange practices in chemistry. PLoS One.

[CR88] King RA, Noakes M, Bird AR, Morell MK, Topping DL (2008). An extruded breakfast cereal made from a high amylose barley cultivar has a low glycemic index and lower plasma insulin response than one made from a standard barley. Journal of Cereal Science.

[CR89] Kopelman PG (2000). Obesity as a medical problem. Nature.

[CR90] Kusano M, Tabuchi M, Fukushima A, Funayama K, Diaz C, Kobayashi M, Hatashi N, Tsuchiya YN, Takahashi H, Kamata A, Yamaya T, Saito K (2011). Metabolomics data reveal a crucial role of cystolic glutamine synthetase 1;1 in coordinating metabolic balance in rice. Plant J.

[CR91] Lamberts L, De Bie E, Derycke V, Veraverbeke WS, De Man W, Delcour JA (2006). Effect of processing conditions on color change of brown and milled parboiled rice. Cereal Chemistry.

[CR92] Leal J, Luengo-Fernández R, Gray A, Petersen S, Rayner M (2006). Economic burden of cardiovascular diseases in the enlarged European Union. Eur Heart J.

[CR93] Liang JFB, Han ZL, Han MJ, Nout R, Hamer RJ (2007). Iron, zinc and phytic acid content of selected rice varieties from China. J Sci Food Agric.

[CR94] Liang J, Li Z, Tsuji K, Kazuhiko Nakano MJ, Nout R, Hamer RJ (2008). Milling characteristics and distribution of phytic acid and zinc in long-, medium- and short-grain rice. Journal of Cereal Science.

[CR95] Liotta LA, Stetler-Stevenson WG (1991). Tumor invasion and metastasis: an imbalance of positive and negative regulation. Cancer Research (Supplement).

[CR96] Liu JC, Ockenden I, Truax M, Lott JNA (2004). Phytic acid-phosphorus and other nutritionally important mineral nutrient elements in grains of wild-type and low phytic acid (lpa1-1) rice. Seed Science Research.

[CR97] Lochner J, Rugge B, Judkins D (2006). How effective are lifestyle changes for controlling hypertension?. J Fam Pract.

[CR98] London S (2004). Cardiovascular disease threatens developing countries. Br Med J.

[CR99] Lopez-Ridaura R, Willett WC, Rimm EB, Liu S, Stampfer MJ, Manson JE, Hu FB (2004). Magnesium intake and risk of type 2 diabetes in men and women. Diabetes Care.

[CR100] Lucca P, Hurrell R, Potrykus I (2002). Fighting iron deficiency anemia with iron-rich rice. J Am Coll Nutr.

[CR101] Ma G, Jin Y, Piao J, Kok F, Guusje B, Jacobsen E (2005). Phytate, calcium, iron, and zinc contents and their molar ratios in foods commonly consumed in China. J Agric Food Chem.

[CR102] Manful JT, Swetman AA, Coker RD, Drunis A (2007). Changes in the thiamine and riboflavin contents of rice during artisanal parboiling in Ghana. Tropical Science.

[CR103] Mason J, Bailes A, Beda-Andourou M, Copeland N, Curtis T, Deitchler M, Foster L, Hensley M, Horjus P, Johnson C, Lloren T, Mendez A, Munoz M, Rivers J, Vance G (2005). Recent trends in malnutrition in developing regions: Vitamin A deficiency, anemia, iodine deficiency, and child underweight. Food Nutr Bull.

[CR104] McCouch SR, Teytelman L, Xu YB, Lobos KB, Clare K, Walton M, Fu BY, Maghirang R, Li ZK, Xing YZ, Zhang QF, Kono I, Yano M, Fjellstrom RG, DeClerck G, Schneider D, Cartinhour S, Ware D, Stein L (2002). Development and mapping of 2240 new SSR markers for rice (Oryza sativa L.). DNA Res.

[CR105] McCouch SR, Zhao K, Wright M, Tung C-W, Ebana K, Thomson MJ, Reynolds A, Wang D, Geneviev deClerck M, Liakat A, Anna M, McClung Georgia E, Carlos D, Bustamante (2010). Development of genome-wide SNP assays for rice. Breeding Science.

[CR106] Misra A, Singhal N, Khurana L (2010). Obesity, the metabolic syndrome, and type 2 diabetes in developing countries: Role of dietary fats and oils. J Am Coll Nutr.

[CR107] Most MM, Tulley R, Morales S, Lefevre M (2005). Rice bran oil, not fiber, lowers cholesterol in humans. Am J Clin Nutr.

[CR108] Munter de Jeroen SL, Frank BHu, Franz DSM, van Rob M, Dam (2007). Whole grain, bran, and germ Intake and risk of type 2 diabetes: a prospective cohort study and systematic review. PLoS Med.

[CR109] Murray-Kolb LE, Takaiwa F, Goto F, Yoshihara T, Theil EC, Beard JL (2002). Transgenic rice is a source of iron for iron-depleted rats. J Nutr.

[CR110] Nakamura H (1966). Effect of gamma-oryzanol on hepatic cholesterol biosynthesis and faecal excretion of cholesterol metabolites. Radioisotopes.

[CR111] Nanri A, Mizoue T, Noda M, Takahashi Y, Kato M, Inoue M, Tsugane S (2010). Rice intake and type 2 diabetes in Japanese men and women: the Japan Public Health Center-based Prospective Study. Am J Clin Nutr.

[CR112] Nesaretnam K, Meganathan (2011). Tocotrienols: inflammation and cancer. Annals of the New York Acadamy of Science.

[CR113] Nicolosi RJ, Austrian LM, Mark Hegsted D (1991). Rice bran oil lowers serum total and low density lipoprotein cholesterol and apo B levels in nonhuman primates. Atherosclerosis.

[CR114] Nielsen MM, Hansen Å (2008). Rapid high performance liquid chromatography determination of tocopherols and tocotrienols in cereals. Cereal Chemistry.

[CR115] Nisak MY, Barakatun AR, Talib AK, Norimah HG, Azmi KN (2010). Improvement of dietary quality with the aid of a low glycemic index diet in Asian patients with type 2 diabetes mellitus. J Am Coll Nutr.

[CR116] Nugent R (2008). Chronic diseases in developing countries. Ann N Y Acad Sci.

[CR117] Nunes SG, Gomes JC, Cruz R, Jordan CP (1991). Mineral enrichment of rice with hydrothermal treatments/mineral enrichement hidrothermal during processing of rice. Brazilian Archives of Biology and Technology.

[CR118] Oikawa A, Matsuda F, Kusano M, Okazaki Y, Saito K (2008). Rice metabolomics. Rice.

[CR119] Oki T, Masuda M, Nagai S, Take'ichi M, Kobayashi M, Nishiba Y, Sugawara T, Suda I, Sato T (2004). Radical-scavenging activity of red and black rice. Rice is life: scientific perspectives for the 21st century. Proceedings of the World Rice Research Conference Los Banos (Philippines).

[CR120] Otegbayo BO, Osamuel F, Fashakin JB (2001). Effect of parboiling on physico-chemical qualities of two local rice varieties in nigeria. The Journal of Food Technology in Africa.

[CR121] Packer L, Weber SU, Rimbach G (2001). Molecular aspects of alpha-tocotrienol antioxidant action and cell signalling. J Nutr.

[CR122] Panchan K, Naivikul O (2009). Effect of pre-germination and parboiling on brown rice properties. Asian Journal of Food Agriculture Industries.

[CR123] Panlasigui LN, Thompson LU (2006). Blood glucose lowering effects of brown rice in normal and diabetic subjects. International Journal of Food Science and Nutrition.

[CR124] Parashar UD, Hummelman EG, Bresee JS, Miller MA, Glass RI (2003). Global illness and deaths caused by rotavirus disease in children. Emerging Infectious Diseases journal.

[CR125] Park Y, David J, Hunter DS, Bergkvist L, Berrino F, van den Piet A, Brandt JE, Buring GA, Colditz Jo L, Freudenheim CS, Fuchs EG, Alexandra Goldbohm R, Graham S, Harnack L, Anne M, Hartman DR, Jacobs IK, Krogh V, Michael F, Leitzmann ML, McCullough AB, Miller PP, Thomas E, Rohan AS, Walter C, Willett AW, Zeleniuch-Jacquotte A, Shumin M, Zhang, Stephanie A, Smith-Warner (2005). Dietary fiber intake and risk of colorectal cancer. J Am Med Assoc.

[CR126] Parker RA, Pearce BC, Clark RW, Gordon DA, Wright JJ (1993). Tocotrienols regulate cholesterol production in mammalian cells by post-transcriptional suppression of 3-hydroxy-3-methylglutaryl-coenzyme A reductase. J Biol Chem.

[CR127] Pedersen B, Knudsen KE, Eggum BO (1989). Nutritive value of cereal products with emphasis on the effect of milling. World Rev Nutr Diet.

[CR128] Pena B, Yelitza J, Frank P, Mario T (2008). Zinc sérico en menores de 15 años de una comunidad rural del estado Lara. Annal Venezuelan Nutrition.

[CR129] Pins JJ, Geleva D, Keenan JM, Frazel CO, Connor PJ, Cherney LM (2002). Do whole-grain oat cereals reduce the need for antihypertensive medications and improve blood pressure control?. J Fam Pract.

[CR130] Prasad AS (2003). Zinc deficiency. Br Med J.

[CR131] Prasad AS, Miale AJ, Farid Z, Sandstead H, Schulert A (1963). Zinc metabolism in patients with syndrome of iron deficiency anemia, hepatosplenomyaly, dwarfism and hypogonadism. J Lab Clin Med.

[CR132] Qu LQ, Yoshihara T, Ooyama A, Goto F, Takaiwa F (2005). Iron accumulation does not parallel the high expression level of ferritin in transgenic rice seeds. Planta.

[CR133] Qureshi AA, Basil A, Bradlow WA, Salser L, Brace D (1997). Novel tocotrienols of rice bran modulate cardiovascular disease risk parameters of hypercholesterolemic humans. J Nutr Biochem.

[CR134] Qureshi AA, Sami SA, Salser WA, Khan FA (2002). Dose-dependent suppression of serum cholesterol by tocotrienol-rich fraction (TRF25) of rice bran in hypercholesterolemic humans. Atherosclerosis.

[CR135] Ramakrishnan TV, Francis FJ (1979). Stability of carotenoids in model aqueous systems. Journal of Food Quality.

[CR136] Ranawana DV, Henry CJK, Lightowler HJ, Wang D (2009). Glycaemic index of some commercially available rice and rice products in Great Britain. Int J Food Sci Nutr.

[CR137] Rasool AHG, Wong AR (2007). Tocotrienol rich vitamin E: A review of clinical studies. International Medical Journal.

[CR138] Reiner Z, Tedeschi-Reiner E, Romić Z (2005). Effects of rice policosanol on serum lipoproteins, homocysteine, fibrinogen and C-reactive protein in hypercholesterolaemic patients. Clin Drug Investig.

[CR139] Rukmini C, Raghuram TC (1991). Nutritional and biochemical aspects of the hypolipidemic action of rice bran oil: a review. J Am Coll Nutr.

[CR140] Ryan EP, Heuberger AL, Weir TL, Barnett B, Broeckling CD, Prenni JE (2011). Rice bran fermented with Saccharomyces boulardii generates novel metabolite profiles with bioactivity. J Agric Food Chem.

[CR141] Sakamoto S, Hayashi T, Hayashi K, Murai F, Hori M, Kimoto K, Murakami K (2007). Pre-germinated brown rice could enhance maternal mental health and immunity during lactation. Eur J Nutr.

[CR142] Sato S, Soga T, Nishioka T, Tomita M (2004). Simultaneous determination of the main metabolites in rice leaves sing capillary electrophoresis mass pectrometry and capillary electrophoresis diode array etection. Plant J.

[CR143] Sato S, Arita M, Soga T, Nishioka T, Tomita M (2008). Time-resolved metabolomics reveals metabolic modulation in rice foliage. BMC Syst Biol.

[CR144] Sato S, Fukumura K, Nishiyama A, Kanamoto I, Inoue Y, Konishi T (2010). Glycemic index and glucose utilization of rice vermicelli in healthy subjects. Biol Pharm Bull.

[CR145] Schaffer S, Pallauf J, Krawinkel MB (2004). Impact of feeding high-iron rice on plasma iron, hemoglobin and red blood cell variables of early-weaned piglets—A pilot study. Ann Nutr Metab.

[CR146] Schatzkin A, Mouw T, Park Y, Amy F, Subar VK, Hollenbeck A, Michael F, Leitzmann F, Thompson E (2007). Dietary fiber and whole-grain consumption in relation to colorectal cancer in the NIH-AARP diet and health study. Am J Clin Nutr.

[CR147] Seal CJ, Jones AR, Whitney AD (2006). Whole grains uncovered. Nutrition Bulletin.

[CR148] Sen CK, Khanna S, Roy S (2007). Tocotrienols in health and disease: the other half of the natural vitamin E family. Mol Aspects Med.

[CR149] Shallan MA, El-Beltagi HS, Mona AM, Amera TM (2010). Chemical evaluation of pre-germinated brown rice and whole grain rice bread. Electronic Journal of Environmental, Agricultural and Food Chemistry.

[CR150] Shaw JE, Sicree R, Zimmet P (2010). Global estimates of the prevalence of diabetes for 2010 and 2030. Diabetes Res Clin Pract.

[CR151] Shaw, Julia G, Jennifer F, Friedman (2011). Iron deficiency anemia: focus on infectious diseases in lesser developed countries. Anemia, Review Article.

[CR152] Shen Y, Jin L, Xiao P, Yan L, Bao J-S (2009). Total phenolics, flavonoids, antioxidant capacity in rice grain and their relations to grain color, size and weight. Journal of Cereal Science.

[CR153] Lee S, Jeon Un S, Lee Seung J, Kim Y-K, Persson Daniel P, Soren H, Schjørring Jan K, Kakei Y, Masuda H, Nishizawa Naoko K, An G (2009). Iron fortification of rice seeds through activation of the nicotianamine synthase gene. Proceedings of the National Academy of Sciences.

[CR154] Shimizu T (2011). Health claims and scientific substantiation of functional foods – Japanese regulatory system and the international comparison. European Food and Feed Law Review.

[CR155] Slavin JL, Martini MC, Jacobs DR, Marquart L (1999). Plausible mechanisms for the protectiveness of whole grains. Am J Clin Nutr.

[CR156] Statistics (2006). Non-Communicable Diseases Surveillance.

[CR157] Statistics (2010). Statistics On Causes Of Death.

[CR158] Stewart D, Louise V, Shepherd T, Robert D, Hall P, Fraser D (2011). Crops and tasty, nutritious food – how can metabolomics help?. In Annual Plant Reviews.

[CR159] Storck CR, da Silva LP, Fagundes CAA (2005). Categorizing rice cultivars based on differences in chemical composition. Journal of Food Composition and Analysis.

[CR160] Sun Q, Spiegelman D, van Dam RM, Holmes MD, Malik VS, Willett WC, Hu FB (2010). White rice, brown rice, and risk of type 2 diabetes in US men and women. Arch Intern Med.

[CR161] Sundstrom FJ, Williams J, Van A, Deynze, Bradford KJ (2002). Identity preservation of agricultural commodities. Agricultural Biotechnology in California Series.

[CR162] Sung HG, Shin HT, Ha JK, Lai HL, Cheng KJ, Lee JH (2005). Effect of germination temperature on characteristics of phytase production from barley. Bioresour Technol.

[CR163] Tamura T, Goldenberg RL (1996). Zinc nurture and pregnancy outcome. Nutr Res.

[CR164] Tang G, Qin J, Dolnikowski GG, Russel RM, Grusak MA (2009). Golden Rice is an effective source of vitamin A. Am J Clin Nutr.

[CR165] Tarpley L, Roessner U, Upadhyaya NM (2007). Metabolomics: enabling systems-level phenotyping in rice functional genomics. Rice Functional Genomics: Challenges.

[CR166] Tian S, Nakamura K, Kayahara H (2004). Analysis of phenolic compounds in white rice. Brown Rice, and Germinated Brown Rice Journal of Agricultural & Food Chemistry.

[CR167] Topping DL (2007). Cereal complex carbohydrates and their contribution to human health. Journal of Cereal Science.

[CR168] Topping DL, Clifton PM (2001). Short-chain fatty acids and human colonic function: Roles of resistant starch and nonstarch polysaccharides. Physiol Rev.

[CR169] Topping D, Fukushima M, Bird AR (2003). Resistant starch as a prebiotic and synbiotic: state of the art. Proc Nutr Soc.

[CR170] Tucker G (2003). Nutritional enhancement of plants. Curr Opin Biotechnol.

[CR171] Tung C-W, Zhao K, Mark Wright M, Ali JJ, Kimball J, Tyagi W, Thomson M, McNally K, Leung H, Kim H, Ahn S-N, Reynolds A, Scheffler B, Eizenga G, McClung A, Bustamante C, McCouch S (2011). Development of a research platform for dissecting phenotypeâ€“genotype associations in rice (<i > Oryza</i > spp.). Rice.

[CR172] Udomkesmalee E, Dhanamitta S, Yhoung-Aree J, Rojroongwasinkul N, Smith JC (1990). Biochemical evidence suggestive of suboptimal zinc and vitamin A status in schoolchildren in Northeast Thailand. American Journul of Clinical Nutrition.

[CR173] Usuki SY, Ito Y, Morikawa K, Kise M, Toyohiko A, Rivner M, Yu R (2007). Effect of pre-germinated brown rice intake on diabetic neuropathy in streptozotocin-induced diabetic rats. Nutr Metab.

[CR174] Varady KA, Wang Y, Jones PJ (2003). Role of policosanols in the prevention and treatment of cardiovascular disease. Nutr Rev.

[CR175] Vasconcelos M, Datta K, Oliva N, Khalekuzzaman M, Torrizo L, Krishnan S, Oliveira M, Goto F, Datta SK (2003). Enhanced iron and zinc accumulation in transgenic rice with the ferritin gene. Plant Sci.

[CR176] Verhoeven HA, Jonker H, De Vos RCH, Hall RD, Hardy NG, Hall RD (2011). Measuring plant volatiles. Methods for Plant metabolomics.

[CR177] Verschoyle RD, Greaves P, Cai H, Edwards RE, Steward WP, Gescher AJ (2007). Evaluation of the cancer chemopreventive efficacy of rice bran in genetic mouse models of breast, prostate and intestinal carcinogenesis. Br J Cancer.

[CR178] Villegas R, Liu S, Gao YT, Yang G, Li H, Zheng W, Shu XO (2007). Prospective study of dietary carbohydrates, glycemic index, glycemic load, and incidence of type 2 diabetes mellitus in middle-aged Chinese women. Arch Intern Med.

[CR179] Virk P, Gerard B (2007). Biofortified rice – towords combating human micronutrient deficiencies.

[CR180] Wang RM, Yang XE, He CX (2004). Genetic analysis on agronomic traits related to zinc efficiency in lowland rice. Journal of Plant Nutrition.

[CR181] Wang L, Michael Gaziano J, Liu S, JoAnn E, Manson JE, Howard B, Sesso D (2007). Whole- and refined-grain intakes and the risk of hypertension in women. Am J Clin Nutr.

[CR182] Welch RM, Graham RD (2004). Breeding for micronutrients in staple food crops from a human nutrition perspective. J Exp Bot.

[CR183] West KP (2002). Extent of vitamin A deficiency among preschool children and women of reproductive age. J Nutr.

[CR184] WHO (2001). World health report 2001, health systems: improving performance.

[CR185] WHO (2002). Health situation in the South East Asia Region 1998–2000.

[CR186] WHO (2008). Worldwide prevalence of anaemia 1993–2005.

[CR187] Wild S, Roglic G, Green A, Sicree R, King H (2004). Global prevalence of diabetes. Diabetes Care.

[CR188] Williams MT, Hord NG (2005). The role of dietary factors in cancer prevention: beyond fruits and vegetables. Nutr Clin Pract.

[CR189] Williams P, Brand Miller J, Fitzgerald M (2005). Project 4505 GI of Rice.

[CR190] Wilson TA, Nicolosi RJ, Woolfrey B, Kritchevsky D (2007). Rice bran oil and oryzanol reduce plasma lipid and lipoprotein cholesterol concentrations and aortic cholesterol ester accumulation to a greater extent than ferulic acid in hypercholesterolemic hamsters. J Nutr Biochem.

[CR191] Wright M, Tung C-W, Zhao K, Reynolds A, McCouch SR, Bustamante CD (2010). ALCHEMY: a reliable method for automated SNP genotype calling for small batch sizes and highly homozygous populations. Bioinformatics (Oxford).

[CR192] Xu Z, Samuel Godber J (1999). Purification and identification of components of gamma-oryzanol in rice bran oil. J Agric Food Chem.

[CR193] Yamakawa H, Hakata M (2010). Atlas of rice grain filling-related metabolism under high temperature: Joint analysis of metabolome and transcriptome demonstrated inhibition of starch accumulation and induction of amino acid accumulation. Plant Cell Physiol.

[CR194] Yoon KH, Lee JH, Kim JW, Cho JH, Choi YH, Ko SH, Zimmet P, Son HY (2006). Epidemic obesity and type 2 diabetes in Asia. Lancet.

[CR195] You D, Wardlaw T, Salama P, Jones G (2010). Levels and trends in under-5 mortality, 1990–2008. Lancet.

[CR196] Yunus AM, Sherina MS, Nor Afiah MZ, Rampal L, Tiew KH (2004). Prevalence of cardiovascular risk factors in a rural Community in Mukim Dengkil, Selangor. Malaysian Journal of Nutrition.

[CR197] Zaidi AKM, Awasthi S, Janaka H, De S (2004). Burden of infectious diseases in South Asia. Br Med J.

[CR198] Zhang G, Vasanti Malik S, Pan A, Kumar S, Holmes M, Donna Spiegelman X, Lin, Frank Hu B (2010). Substituting brown rice for white rice to lower diabetes risk: A focus-group study in Chinese adults. J Am Diet Assoc.

[CR199] Zheng L, Cheng Z, Ai C, Jiang X, Bei X, Zheng Y, Glahn RP, Welch RM, Miller DD, Lei XG, Shou H (2010). Nicotianamine, a novel enhancer of rice iron bioavailability to humans. PLoS One.

[CR200] Zhou JM, Ibrahim RK (2010). Tricin—a potential multifunctional nutraceutical. Phytochemistry Reviews.

[CR201] Zhou Z, Robards K, Helliwell S, Blanchard C (2004). The distribution of phenolic acids in rice. Food Chem.

[CR202] Zhou J, Ma C, Xu H, Yuan KL, Lu X, Zhu Z, Wu YN, Xu GW (2008). Metabolic profiling of transgenic rice with cryIAc and sck genes: an evaluation of unintended effects at metabolic level by using GC-FID and GC-MS. J Chromatogr B.

