# Supplementary figures and images for: The potential of rice to offer solutions for malnutrition and chronic diseases
Source: Rice (N Y). 2012 Jul 2;5:16. doi: 10.1186/1939-8433-5-16 (PMC4883736; doi:10.1186/1939-8433-5-16)

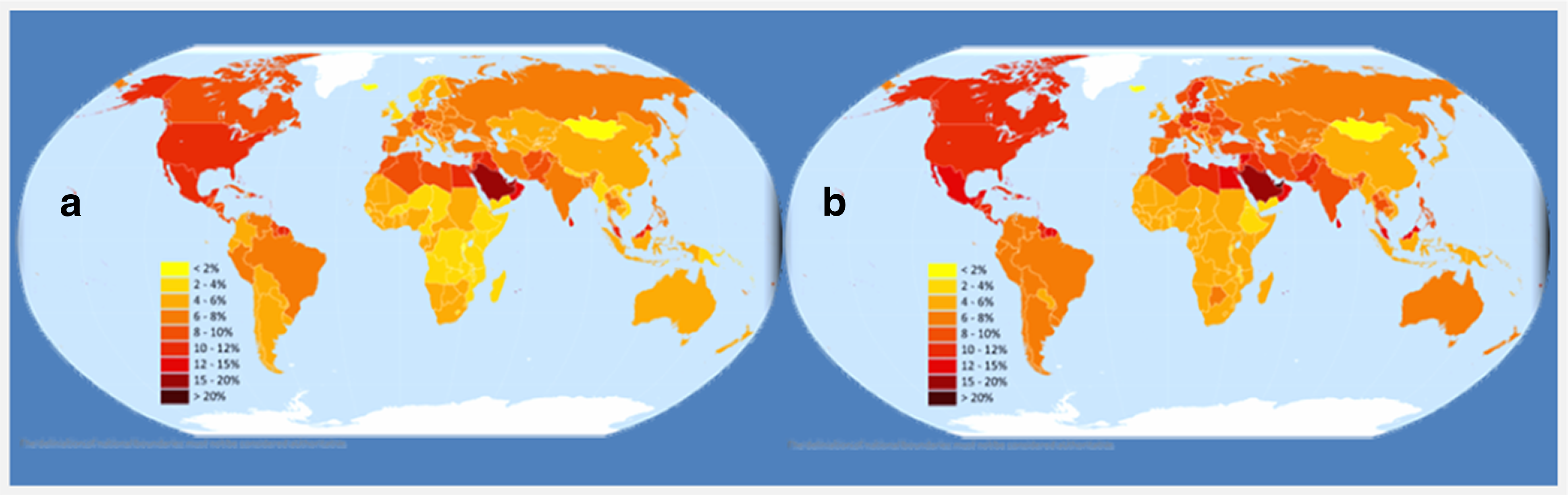

Supplement: Supplementary file 1 — Authors’ original file for figure 1 [file 12284_2011_28_MOESM1_ESM.tiff]

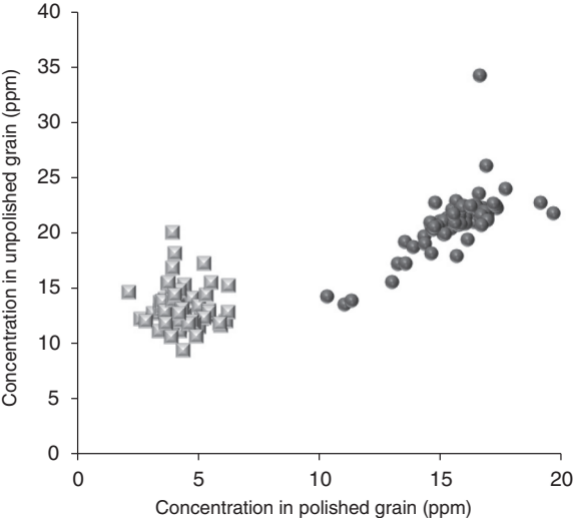

Supplement: Supplementary file 2 — Authors’ original file for figure 2 [file 12284_2011_28_MOESM2_ESM.pdf]

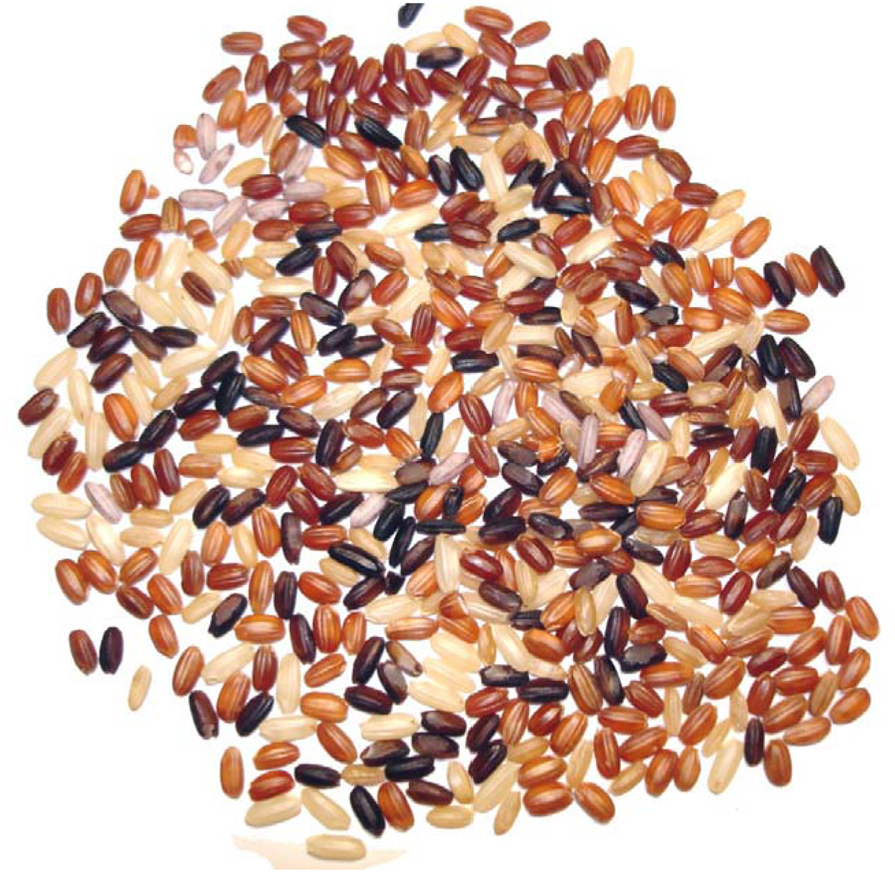

Supplement: Supplementary file 3 — Authors’ original file for figure 3 [file 12284_2011_28_MOESM3_ESM.tiff]
